# Clinical, Biochemical and Genetic Variables Associated With Metabolic Syndrome in Patients With Schizophrenia Spectrum Disorders Using Second-Generation Antipsychotics: A Systematic Review

**DOI:** 10.3389/fpsyt.2021.625935

**Published:** 2021-03-29

**Authors:** Marius H. Sneller, Nini de Boer, Sophie Everaars, Max Schuurmans, Sinan Guloksuz, Wiepke Cahn, Jurjen J. Luykx

**Affiliations:** ^1^Faculty of Biomedical Sciences, Utrecht University, Utrecht, Netherlands; ^2^Department of Psychiatry, University Medical Center Utrecht Brain Center, University Medical Center Utrecht, Utrecht University, Utrecht, Netherlands; ^3^Faculty of Medicine, Utrecht University, Utrecht, Netherlands; ^4^Department of Psychiatry and Neuropsychology, School for Mental Health and Neuroscience, Maastricht University Medical Centre, Maastricht, Netherlands; ^5^Department of Psychiatry, Yale University School of Medicine, New Haven, CT, United States; ^6^Altrecht Mental Health, Utrecht, Netherlands; ^7^Department of Translational Neuroscience, University Medical Center Utrecht Brain Center, University Medical Center Utrecht, Utrecht University, Utrecht, Netherlands; ^8^GGNet Mental Health, Apeldoorn, Netherlands

**Keywords:** metabolic syndrome, antipsychotics, psychotic spectrum disorder, schizophrenia, systematic review

## Abstract

**Background:** Individuals with severe mental illness experience increased morbidity and mortality compared to the general population. Adverse effects of antipsychotics, including weight gain, may contribute to the development of metabolic syndrome (MetS), which is associated with increased risks of all-cause and cardiovascular disease mortality. We aim to provide a comprehensive overview of clinical, biochemical and genetic factors associated with MetS among patients with schizophrenia spectrum disorders using second-generation antipsychotics (SGA).

**Methods:** A literature search was performed in Pubmed and Embase to identify all cohort studies, cross-sectional studies and clinical trials investigating associations with MetS in patients with schizophrenia spectrum disorders using SGAs. We extracted and enumerated clinical, biochemical and genetic factors reported to be associated with MetS. We defined factors associated with MetS as factors being reported as associated with MetS in two or more studies.

**Results:** 58 studies were included in this review (*n* = 12,123). In total, 62 factors were found to be associated with increased risk of MetS. Thirty one out of 58 studies investigated factors that were reported as associated with MetS in two or more studies. With regard to clinical factors, we found gender, higher age, concomitant use of mood stabilizers, higher baseline and current BMI, earlier SGA exposure, higher dose, longer duration of treatment, psychosis and tobacco smoking to be significantly associated with MetS. Furthermore, the biochemical factors hypo-adiponectinemia, elevated levels of C-reactive protein (CRP) and higher white blood cell (WBC) count were identified as factors associated with MetS. Among pharmacogenetic factors, the rs1414334 C-allele of the HTR2C-gene was associated with MetS in patients using SGA.

**Conclusion:** In this systematic review investigating clinical, biochemical and genetic factors associated with MetS in patients using SGAs we found that higher age, higher baseline BMI, higher current BMI and male as well as female gender were positively associated with MetS across all antipsychotics. This study may set the stage for the application of clinical, biochemical and genetic factors to predict the risk of developing MetS in patients using SGAs. Future research is needed to determine which patients using SGAs are at risk to develop MetS in clinical practice.

## Introduction

Patients with psychotic spectrum disorders have a markedly reduced life expectancy compared to the general population. For instance, patients with schizophrenia have a reduced life span of 15–20 years compared to the general population and also have more somatic co-morbidities (1, 2). This is partially due to the development of the metabolic syndrome (MetS). MetS is a combination of risk factors that can lead to increased mortality and morbidity such as cardiovascular disease and diabetes (3). According to The US National Cholesterol Education Programme Adult Treatment Panel III (ATP III), MetS is diagnosed when a person fulfills at least three of the following criteria: waist size of at least 102 cm for males and at least 88 cm for females; triglycerides of at least 150 mg/dl; HDL cholesterol level of <40 mg/dl for males and <50 mg/dl for females; a blood pressure of more than 130 mmHg systolic or 85 mmHg diastolic; and a fasting glucose of more than 100 mg/dl (4). The International Diabetes Federation (IDF) applies similar criteria but requires the presence of an increased, ethnicity specific waist size plus two or more of the abovementioned factors (5).

Rates of MetS vary significantly between populations. Genetic and geographical environmental differences are known to affect metabolic risk factors. For Europeans the age-adjusted rates are 18.4% for men and 14.4% for women while in South Asians the occurrence in men is 28.8 and 31.8% for women, based on the ATP III MetS definition (6). In Japan, the rate of MetS is 14.2% in the general population (7) while in the United States, the age-adjusted weighted prevalence is 34.3% (8). Compared to the general population, patients with schizophrenia have a 2- to 3-fold increased prevalence of MetS varying per country (9, 10). Given the higher prevalence of MetS among these schizophrenia patients, the syndrome poses a greater health risk to this population. It has a significant impact on morbidity and mortality due to the increased risk of diabetes mellitus type 2 (DM2) and cardiovascular disease (3). Explanations for this higher prevalence are a poor diet, cigarette smoking (11, 12), lack of exercise (12), stress and abnormalities in the hypothalamic-pituitary-adrenal axis (13). On top of this, SGA use is associated with metabolic abnormalities and may exacerbate this condition by causing weight gain, glucose and lipid metabolism deregulation (6, 14–18). Antipsychotics can influence metabolic parameters within 2 weeks of treatment (19). However, the existing body of research suggests that the degree of metabolic dysregulations varies considerably between different SGAs (19). Evidence for weight gain was found for clozapine, zotepine, olanzapine, and sertindole, iloperidone, quetiapine, risperidone and paliperidone, and brexpiprazole. SGA are also associated with glucose abnormalities and development of DM2 (9, 20–23). Pillinger et al. (24), performed a large meta-analysis to compare and rank antipsychotics based on their metabolic side-effects and to identify predictors of antipsychotic-induced metabolic dysregulation. Increased baseline weight, male sex, and non-white ethnicity were found to be predictors of susceptibility to antipsychotic induced metabolic change (24). Increase in fasting-glucose was associated with a higher risk of (cardio)vascular disease and was especially evident in olanzapine, zotepine, and clozapine use (24). Furthermore, several studies have demonstrated lipid disturbances following SGA-use (25). Evidence was found that quetiapine, olanzapine, zotepine, and clozapine are negatively correlated with triglyceride alterations (24). Finally, research indicates that patients using SGA have increased cholesterol levels (26), especially in patients using quetiapine, olanzapine, or clozapine (24). Clozapine is the most effective treatment to improve symptom severity and to reduce the risk of recurrent suicidal behavior in patients with schizophrenia or schizoaffective disorder (27, 28). Meanwhile, together with olanzapine, it is also associated with the highest increases in weight, body mass index (BMI) and total cholesterol, suggesting that the greatest metabolic disturbances are caused by the most efficacious antipsychotics (16, 24, 29).

Qualitative research shows that patients have concerns about the negative long-term effects of antipsychotics on their physical appearance and physical health (30, 31). Multiple studies show that patients using antipsychotic medication consider weight gain, possibly leading to overweight and obesity, as one of the most disturbing adverse events and therefore one of the major reasons for non-adherence to therapy (32, 33). Taking this into consideration, elucidating factors that contribute to the occurrence of MetS in specific antipsychotics is useful for clinical practice.

Although the use of SGA is thus clearly associated with increased risk to develop MetS, specific factors that increase this risk have remained largely elusive. Apart from the effects accounted for by lifestyle and antipsychotic medication, research hints at a shared underlying pathophysiology between schizophrenia and cardiovascular disease (34). The high interindividual variability in the occurrence of MetS suggests that genetic factors influence its risk (35). In previous studies, factors associated with MetS among patients with psychotic spectrum disorders who use SGA have been examined (36–38). These factors, however, have been discussed in isolation and findings remain inconclusive. Furthermore, in the analysis by Pillinger et al. (24), MetS was not examined as an outcome measure; only isolated factors (e.g., dislipidemia, hypercholesteremia) were investigated. We chose to perform a systematic review in which we included only studies that took MetS (the combination of risk factors) as an outcome measure. Since people with MetS have a reduced life span of 15–20 years compared to the general population and also have more somatic co-morbidities (39), we reasoned there would be added value in considering MetS as a conglomerate of factors, instead of its isolated components. Finally, in the study of Pillinger et al. (24), limited clinical and biochemical factors and no genetic factors were investigated, which we here expanded on. Thus, we conducted a systematic review of factors associated with MetS during treatment with SGAs (4, 5).

## Methods

### Search Strategy

The systematic review was performed in accordance with the preferred reporting items for systematic reviews and meta-analyses (PRISMA) guidelines (40). Articles were identified through searches in PubMed and Embase from inception until July 25, 2020. Synonyms of the following search terms were used: (schizo^*^ OR psychos^*^) AND (“metabolic syndrome(s)” OR “syndrome X”) AND (antipsychotic OR [generic/branded antipsychotic names]). The search strategy is described extensively in the Appendix (p. 29). We included all cohort studies, cross-sectional studies and clinical trials investigating factors associated with MetS among patients with psychosis spectrum disorders using clozapine, olanzapine, quetiapine, risperidone, aripiprazole, ziprasidone, lurasidone, asenapine, zotepine, and paliperidone (Appendix, p. 1–5).

### Inclusion and Exclusion Criteria

The following studies were included: (i) studies which reported factors associated with MetS; (ii); studies which were cohort studies, cross-sectional studies or clinical trials; (iii) studies that have been written in English or Dutch; (iv) studies with full text availability; (v) studies that were conducted in adult human participants (≥18 years, with no upper age limit) with a diagnosis of psychotic spectrum disorders classified according to DSM-5 criteria (schizophrenia, schizophreniform disorders, schizoaffective disorders, delusional disorders, short-term psychotic disorders and catatonia) and; (vi) studies that investigated the outcome MetS using ATP III, ATP III-A or IDF criteria (4, 5). Presence of MetS in the individual studies was considered only if defined according to one of the following accepted criteria, meaning either the IDF criteria or the National Cholesterol Education Programme's Adult Treatment Panel III criteria (NCEP/ATP III), or the modified IDF and modified NCEP/ATP III criteria with Asian cutoffs for BMI and waist circumference, or the American Heart Association/National Heart, Lung and Blood Institute (AHA/NLHBI) criteria. When the full text of an article was not available through our University library, librarians tried to retrieve the article from other sources. Studies were excluded from the review if they were: (i) animal studies and (ii) reviews/meta-analyses.

### Data Extraction and Reporting of Results

Articles were included or excluded based on title and abstract. In case of doubt, the full text of the articles was screened. The snowball method was used by checking the references of the retrieved articles, including reviews, on potential additional literature for the current review. Article screening and data extraction was performed by S.E., M.S. and M.H.S. Of all included studies author, year of publication, study type, antipsychotics, factors, outcomes, prevalence of MetS, *p*-values, odds ratios (OR), confidence interval (CI), follow-up time, origin of population, sample size (*n*), male/female ratio, mean age and mean duration of SGA treatment were gathered. A factor was considered as significant in this review when authors named it as significant. It was decided not to use *p* = 0.05 as the limit for significance, because the number of factors investigated influences the interpretation of the p-value due to a higher chance of type 1 errors. For our systematic review, we enumerated the clinical, biochemical and genetic factors reported as significantly associated with MetS in the studies as outcome measure. Only factors that were found to be associated with MetS in two or more studies were included. Studies on factors that were not found to be associated, or factors that were found to be associated in only one study, were included in Supplementary Material (see Supplementary Tables 1–3 for study characteristics and Supplementary Tables 4–11 for results on investigated factors). Solely data on subjects with complete metabolic profile is included. Results on associated factors are presented separately for studies investigating a SGA individually and for studies investigating a pooled group of antipsychotics.

### Quality Assessment

The quality of the articles was assessed using the Quality In Prognosis Studies (QUIPS) checklist (41). When using this method, articles are scored on: Study Participation, Study Attrition, Prognostic Factor Measurement, Outcome Measurement, Study Confounding and Statistical Analysis and Reporting by marking them as low risk, uncertain risk and high risk of bias. Study Participation addresses the representativeness of the study sample for the source population. Studies with low participation rates, considerable differences in age and sex distribution or very selective eligibility criteria were considered to have a high risk of bias, while studies with high participation and a study sample comparable to the entire population had a low risk of bias. Study Attrition addresses the possible bias with regards to participants who do not complete the study. Studies with high withdrawal rates and inconclusive follow-up information have a high risk of bias, while studies with complete follow-up data, or evidence of participants missing at random, have a low risk of bias. The Prognostic Factor Measurement and Outcome Measurement domain addresses the clarity of outcome definition, the validity and reliability of measurement and the similarity of measurements. Differential measurements between groups are considered to contribute to a high risk of bias, while similarity and reliability of measurements are considered to have a low risk of bias. Study Confounding addresses the risk of bias with regards to the possibility of confounding factors that might contribute to the outcome. Adequate measurement of potential confounding variables and correction for these factors lowers the risk of bias. Finally, the Statistical Analysis and Reporting domain addresses the suitability of the statistical analysis and the completeness of reporting in a study. The risk of bias in this domain is considered low if the analysis is appropriate for the data, statistical requirements are met and all primary outcomes are reported. Thus, generally we used the QUIPS checklist to evaluate the risk of bias. Studies that scored high risk of bias in more than four categories were excluded from our systematic review.

## Results

### Search Results

**Figure 1:**
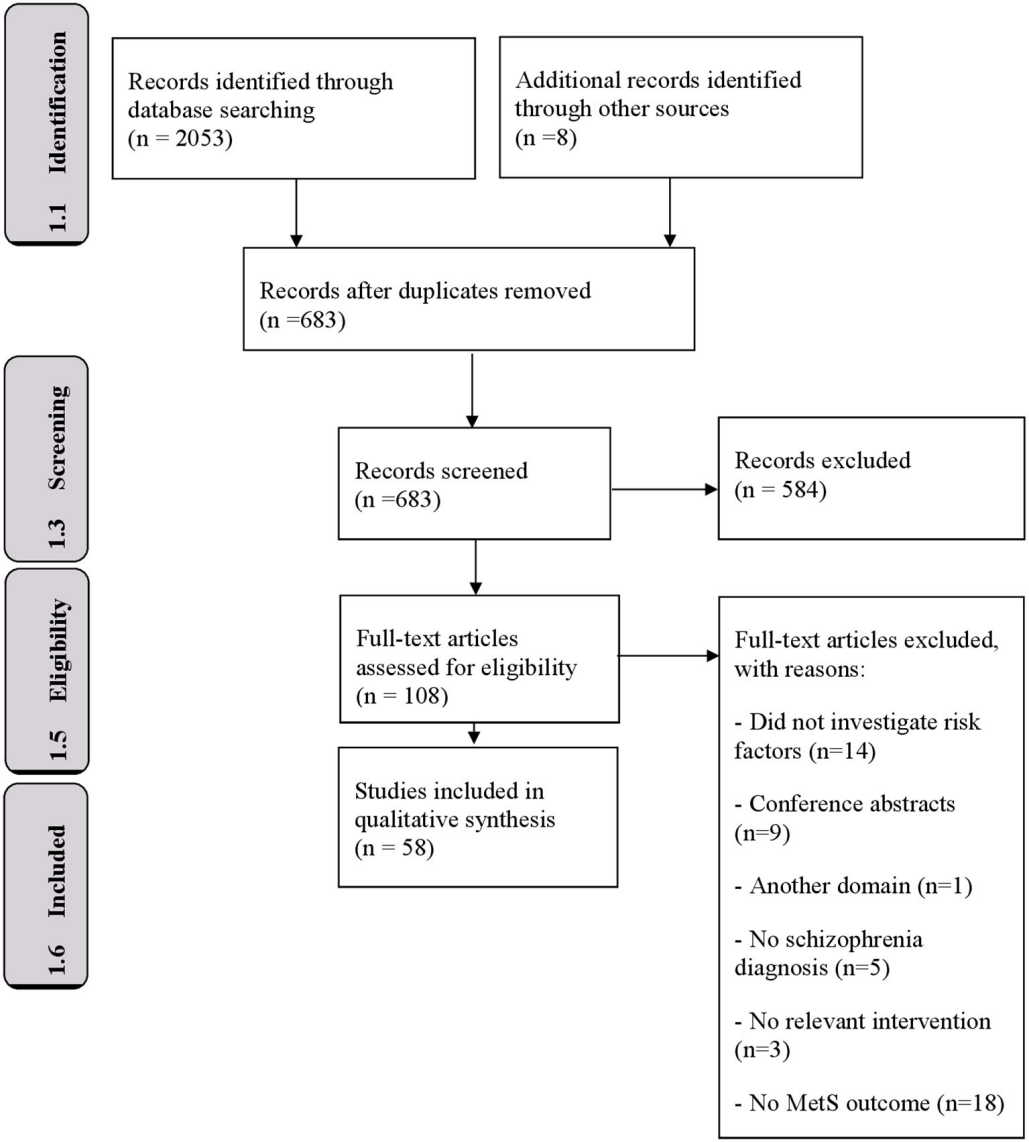
Flow diagram of the review selection process.

The study selection process for this systematic review is summarized in Figure 1. The initial search identified 2,053 research articles. The snowball method resulted in an additional 8 articles. After removing duplicates, 683 unique articles were screened for suitability for inclusion, utilizing the search criteria defined above, by reading the titles and abstracts. Of these, 108 articles were extracted for further evaluation to assess the full-text of these articles for potential eligibility. The references of these full-text articles were also scrutinized to identify additional eligible publications. Fifty articles were excluded because they: did not provide data on schizophrenia diagnosis (*n* = 5), did not perform a relevant intervention (*n* = 3), did not use MetS as outcome measure (*n* = 18), were in a different domain (*n* = 1), did not investigate risk factors (14) or solely provided conference abstracts (*n* = 9). These studies reported *p*-values, odds ratios and F-values (Tables 2, 3). All steps resulted in 58 studies meeting the selection criteria.

### Study Characteristics

The 58 included studies were made up of eight cohort studies, 44 cross-sectional studies, five case-control studies and one clinical trial. Of the 58 studies, 26 investigated a single SGA and 32 multiple SGAs. The studies were conducted in: China (11), United States (9), Taiwan (7), the Netherlands (5), Korea (3), India (2), Croatia (2), Turkey (2), Serbia (2), Ireland (1), Australia (1), Venezuela (1), Brazil (1), Thailand (1), Sudan (1), Sweden (1), Finland (1), United Kingdom (1), Germany (1), Malaysia (1), Denmark (1), Romania (1), Chile (1), and Italy (1). Thus, 23 out of 58 studies (40%) had been conducted in low- and middle-income countries (2 and 21 studies, respectively). Twenty-seven of the studies were performed in Asian countries. The number of participants per study varied from 24 to 621. The total number of participants was 12.123 (excluding overlap of sample size). The most commonly studied antipsychotics were: clozapine (5,739 participants); olanzapine (2,081 participants), risperidone (1,875 participants), quetiapine (233 participants), aripiprazole (110 participants) and paliperidone (79 subjects). Two hundred and sixty-four participants received polytherapy. Regarding the studies that investigated single SGAs, clozapine was the most prevalent (21 studies), followed by olanzapine (six studies), risperidone (one study) and aripiprazole (one study). The mean duration of SGA treatment ranged from 10 to 209 months. The MetS prevalence ranged from 28.4 to 64%. Forty-four of the 58 studies were cross-sectional, which limits the ability to draw valid conclusions about possible causality because the presence of risk factors and outcomes are measured simultaneously. See Supplementary Tables 1–3 for the study characteristics of all included studies.

### Quality Assessment

Quality assessment was conducted based on the reporting and methodological quality of the studies. There was a high risk of bias regarding study participation in 18 studies, 16 had an unclear risk of bias and 20 a low risk of bias regarding study participation (Table 1). Most of the studies had a low risk of bias with regards to study attrition. Only four were considered to have a high risk of bias regarding study attrition, while three had an uncertain risk of bias. The domain of prognostic factor measurement was found to have a high risk of bias in three studies, while the other 55 were not considered to have a risk of bias in this domain. The outcome measurement was clearly defined and established in all studies and was therefore considered to have low risk of bias in this category. Most of the studies were cross-sectional studies that, due to the nature of their design, were more prone to confounding. The risk of bias due to study confounding was therefore considered to be high in 45 of the studies; seven had an uncertain risk of bias, while six had a low risk of bias. Finally, the risk of bias regarding statistical analysis and reporting was considered high in 13 studies, while 45 were considered to have a low risk of bias. Thus, generally studies that had high risk of bias in three or more of the QUIPS categories were excluded from the analysis. None of the identified studies had a high risk of bias in more than four categories, therefore all 58 were included in this systematic review.

**Table 1:** Quality assessment of the included studies.

	**Study participation**	**Study attrition**	**Prognostic factor measurement**	**Outcome measurement**	**Study confounding**	**Statistical analysis and reporting**
Mohamed et al. (42)	**?**	**+**	**+**	**+**	**+**	**+**
Ventriglio et al. (43)	**?**	**+**	**+**	**+**	**–**	**+**
Iruretagoyena et al. (44)	**–**	**+**	**+**	**+**	**–**	**+**
Dehelean et al. (45)	**–**	**+**	**+**	**+**	**–**	**+**
Chen et al. (46)	**+**	**+**	**+**	**+**	**–**	**+**
Puangpetch et al. (47)	**+**	**+**	**+**	**+**	**–**	**+**
Pinto et al. (48)	**+**	**+**	**+**	**+**	**–**	**–**
Lu et al. (49)	**+**	**+**	**+**	**+**	**–**	**+**
Larsen et al. (50)	**–**	**+**	**+**	**+**	**–**	**+**
Chen et al. (51)	**–**	**+**	**+**	**+**	**–**	**+**
Zhang et al. (52)	**–**	**+**	**+**	**+**	**–**	**+**
Zhang et al. (53)	**?**	**+**	**+**	**+**	**–**	**+**
Kraal et al. (54)	**?**	**–**	**+**	**+**	**–**	**+**
Yang et al. (55)	**–**	**+**	**–**	**+**	**–**	**+**
Yang et al. (56)	**–**	**+**	**+**	**+**	**–**	**+**
Saatcioglu et al. (57)	**?**	**+**	**?**	**+**	**–**	**+**
Popovic et al. (58)	**?**	**+**	**+**	**+**	**–**	**+**
Yang et al. (59)	**–**	**+**	**+**	**+**	**+**	**–**
Popovic et al. (60)	**?**	**?**	**+**	**+**	**?**	**+**
Lin et al. (61)	**–**	**+**	**+**	**+**	**–**	**+**
Zhang et al. (62)	**–**	**+**	**+**	**+**	**?**	**+**
Roffeei et al. (63)	**?**	**+**	**+**	**+**	**–**	**+**
Zhang et al. (53)	**–**	**+**	**+**	**+**	**–**	**+**
Miller et al. (64)	**–**	**+**	**+**	**+**	**–**	**–**
Lott et al. (65)	**–**	**+**	**+**	**+**	**–**	**–**
Liou et al. (66)	**–**	**+**	**+**	**+**	**?**	**+**
Lee et al. (67)	**+**	**?**	**+**	**+**	**?**	**+**
Chen et al. (68)	**–**	**+**	**+**	**+**	**?**	**+**
Risselada et al. (69)	**–**	**+**	**+**	**+**	**–**	**–**
Liou et al. (70)	**–**	**+**	**+**	**+**	**–**	**+**
Grover et al. (71)	**+**	**+**	**+**	**+**	**–**	**+**
Fernandez et al. (72)	**–**	**+**	**+**	**+**	**–**	**+**
Ellingrod et al. (73)	**?**	**?**	**+**	**+**	**–**	**+**
Lee et al. (74)	**–**	**+**	**+**	**+**	**–**	**–**
Kuzman et al. (75)	**+**	**+**	**+**	**+**	**–**	**–**
Kraemer et al. (76)	**–**	**–**	**+**	**+**	**–**	**–**
Kang et al. (77)	**?**	**+**	**+**	**+**	**–**	**+**
Grover et al. (78)	**?**	**+**	**+**	**+**	**–**	**+**
Van Winkel et al. (79)	**+**	**+**	**+**	**+**	**–**	**+**
Fan et al. (80)	+	+	+	+	+	+
Steylen et al. (81)	**+**	**+**	**+**	**+**	**–**	**+**
Patel et al. (19)	**?**	**+**	**–**	**+**	**–**	**+**
Mulder et al. (82)	**+**	**+**	**+**	**+**	**–**	**–**
Medved et al. (83)	**?**	**+**	**+**	**+**	**–**	**+**
Josiassen et al. (84)	**+**	**+**	**–**	**+**	**–**	**–**
Bai et al. (85)	**+**	**–**	**?**	**+**	**–**	**–**
Brunero et al. (86)	**?**	**+**	**+**	**+**	**–**	**+**
Yevtushenko et al. (87)	**+**	**–**	**+**	**+**	**–**	**+**
Ojala et al. (88)	**+**	**+**	**+**	**+**	**+**	**+**
Lee et al. (89)	**–**	**+**	**+**	**+**	**+**	**+**
Ellingrod et al. (90)	**?**	**+**	**+**	**+**	**–**	**–**
Boke et al. (91)	**+**	**+**	**+**	**+**	**+**	**+**
Ahmed et al. (92)	**?**	**+**	**+**	**+**	**–**	**+**
Mulder et al. (93)	**+**	**+**	**+**	**+**	**–**	**+**
Bai et al. (94)	**?**	**+**	**+**	**+**	**–**	**+**
Lamberti et al. (22)	**+**	**+**	**+**	**+**	**–**	**+**
Hägg et al. (95)	**+**	**+**	**+**	**+**	**?**	**+**
Kato et al. (96)	**+**	**+**	**+**	**+**	**?**	**–**

*[–/red], high risk of bias; [?/yellow], uncertain risk of bias; [+/green], low risk of bias*.

### Clozapine

Nine clinical factors were found to be related to MetS (Table 2). Three studies found an association between male gender and MetS in patients treated with clozapine (47, 86, 92). In three other studies of clozapine, an association between female gender and MetS was found (62, 81, 84). The use of concomitant mood stabilizers was reported to be a risk factor for MetS in two studies (81, 85, 94). Bai et al. (94) and Josiassen et al. (84), found higher age at initiation of clozapine treatment to be associated with MetS. Brunero et al. (86), Lamberti et al. (22), and Bai et al. (94), found higher age to be a risk factor associated with MetS. Higher baseline BMI was reported to be associated with MetS in two studies (84, 94). In four other studies, it was shown that higher current BMI is also a risk factor for MetS in patients treated with clozapine (22, 78, 86, 92). Clozapine dose also seems to affect the occurrence of MetS, as was found by Josiassen et al. (84) and Brunero et al. 2009 (86). Finally, the duration of clozapine treatment was found to be associated with MetS in two studies (22, 84).

**Table 2:** Factors associated with MetS in clozapine users reported by ≥2 studies.

**Factor**	**Study/studies**	**MetS prevalence (%)**	**Test statistics reported in the included studies**	***N***
Male gender	(92)	46.6	OR = 11.18 (*P* = 0.013)	84
	(86)	61.6	*P* = 0.009	73
	(47)	36	OR = 4.33 (*P* = 0.02)	50
Female gender	(84) (#1)	64	F = 4.9 (*P* < 0.05)	25
	(62)	43.2	*P* = 0.04	468
	(81)	61	*P* = 0.012	62
Concomitant use of mood stabilizers	(94)	28.4	OR = 2.642 (*P* = 0.041)	188
	(81)	61	*P* = 0.023	62
(Higher) age at initiation of clozapine treatment	(94)	28.4	OR = 1.056 (*P* =0.049)	188
	(84) (#1)	64	Statistical trend	25
(Higher) age	(94)	28.4	*P* =0.009	188
	(86)	61.6	OR = 1.083 (#2) (*P* = 0.007)	73
	(22)	53.8	*P* < 0.001	93
(Higher) baseline BMI	(94)	28.4	OR = 1.226 (*P* < 0.001)	188
	(84) (#1)	64	F = 16.12 (*P* < 0.005)	25
(Higher) current BMI	(92)	46.6	OR = 1.38 (*P* = 0.001)	84
	(78)	47	*P* = 0.001	100
	(22)	53.8	*P* < 0.0001	93
	(86)	61.6	*P* = 0.001	73
Higher clozapine dose	(84)	64	Statistical trend	25
	(86) (#1)	61.6	*P* = 0.03	73
(Longer) clozapine duration	(84) (#1)	64	F = 5.97 (*P* < 0.01)	25
	(22)	53.8	Statistical trend (*P* = 0.06)	93

*#1: Half (8 of 16) of the subjects already met MetS criteria during first-generation antipsychotic treatment*.

*#2: When controlling for the variables of gender, clozapine dose, duration of clozapine treatment, and concomitant use of mood stabilizers and other antipsychotics*.

### Risperidone, Olanzapine, and Aripiprazole

Eight studies investigated the association between clinical, biochemical or genetic factors and the occurrence of MetS in patients treated with either risperidone, olanzapine or aripiprazole. All reported factors were only found to be associated in single studies (Supplementary Tables 6–9).

### Pooled Results of SGAs

The majority of the studies on risk factors associated with MetS analyzed antipsychotics in pooled groups, consisting of several antipsychotics. Sixteen factors were found to be risk factors for MetS (Table 3). Hypo-adiponectinemia was reported to be associated with MetS in two studies (51, 85). Kraemer et al. (76) and Miller et al. (64), investigated the association of CRP in their patient sample and found values of ≥3 mg/L to be correlating with the occurrence of MetS. Higher total WBC count was found to be associated in two studies (64, 80). Three studies reported male gender to be associated with MetS (46, 74, 76), while four studies found female gender to be a risk factor for MetS (54, 71, 89, 91). Several studies reported a higher age of patients treated with antipsychotics to be significantly associated with the development of MetS (43, 46, 55, 73, 74, 85, 87, 91). Grover et al. (71), found age >35 to be associated with MetS. Ethnicity was not found to be a clear risk factor for MetS.

**Table 3:** Results regarding factors which were associated with MetS in pooled SGAs.

**Factor**	**Study/studies**	**Antipsychotics**	**MetS prevalence** ** (%)**	**Test statistics reported in the included studies**	***N***
Hypo-adiponectinemia	(85)	CLO/OLA/RIS	23.8	*P* < 0.0001	567
	(51)	CLO/OLA	33.2	*P* = 0.005	262
CRP ≥ 3 mg/L	(76)	CLO/OLA/RIS	49.6	OR = 2.00, 95% CI = 1.22 – 3.30(*P* = 0.0062)	476
	(64)	CLO/OLA/RIS/ ARI/QUE/ PAL/HAL/ZIP^*^	32.2	*P* = 0.04	59
Higher total WBC count	(64)	CLO/OLA/RIS/ ARI/QUE/PAL/ HAL/ZIP^*^	32.2	*P* = 0.001	59
	(80)	OLA/RIS^*^	53.8	OR = 47.2, 95% CI = 3.4 – 658.7 (*P* = 0.004)	199
Male gender	(74)	OLA/RISP/ARI	31.7	OR = 2.09, 95% CI = 1.49–2.70 (*P* < 0.05)	145
	(46)	CLO/ARI/AMI/ ZIP/HAL	31.2	OR = 1.45, 95% CI = 1.16–4.65(*P* < 0.05)	
	(76)	OLA/RIS/QUE	49.6	OR = 0.56, 95% CI = 0.34 – 0.91 (*P* = 0.0185)	476
Female gender	(74)	OLA/RISP	14.7	OR = 2.914, 95% CI = 1.373 – 4.454 (*P* < 0.01)	75
	(54)	QUE/RISP/ ILO/PAL^*^	41.1	*P* = 0.05	112
	(91)	N/A	32.0	OR = 4.60, 95% CI = 2.20 – 9.64 (*P* = 0.005)	231
	(71)	CLO/OLA/RIS/ QUE	43.6	OR = 1.81, 95% CI = 1.07 – 3.08 (*P* = 0.027)	227
Higher age	(74)	OLA/RISP/ARI	31.7	*P* = 0.02	145
	(43)	CLO/OLA/RIS/ ARI/QUE/ PAL/HAL	31.8	OR = 1.03, 95% CI = 1.01 −1.07 (*P* = 0.029)	151
	(46)	CLO/ARI/HAL/ AMI/ZIP	31.2	OR= 1.03, 95% CI = 0.98 −1.09 (*P* < 0.05)	157
	(73)	CLO/OLA/RIS/QUE/PAL	41	*P* < 0.001	237
	(85)	CLO/OLA/RIS	23.8	*P* = 0.007	567
	(55)	OLA/RIS^*^	37.8	OR = 1.939, 95% = 1.078 – 3.485 (*P* = 0.012)	357
	(91)	N/A	32.0	*P* = 0.026	231
	(87)	CLO/OLA/RIS^*^	38.3	*P* = 0.003	120
Age > 35	(71)	CLO/OLA/RIS/ QUE	43.6	OR = 3.37, 95% CI = 1.94 – 5.86 (*P* < 0.001)	227
(Higher) baseline BMI	(85)	CLO/OLA/RIS	23.8	*P* = 0.007	567
(Higher) current BMI	(83)	OLA/RISP	27	95% CI = 1.201–1.686(*P* < 0.001)	40
	(57)	CLO/OLA^*^	42.2	OR = 1.389, 95% CI = 1.210 – 1.595(*P* = 0.018)	116
	(95)	CLO/OLA^*^	34.6	*P* < 0.001	269
(Higher) BMI increase after initiation of antipsychotic treatment	(85)	CLO/OLA/RIS	23.8	*P* = 0.007	567
BMI > 25	(71)	CLO/OLA/RIS/QUE	43.6	OR = 5.64 (*P* < 0.001)	227
BMI > 24	(61)	CLO/OLA/RIS/ ARI/QUE/ ZOT/AMI/ZIP	23.7	OR = 6.084, 95% CI = 3.207–11.540 (*P* < 0.001)	329
	(55)	OLA/RIS^*^	37.8	OR = 3.999, 95% CI = 2.482–6.442 (*P* < 0.001)	357
(Higher) dose	(89)	OLA/RISP	14.7	*P* < 0.01	75
	(43)	CLO/OLA/RIS/ ARI/QUE/ PAL/HAL	31.8	OR = 1.003, 95% CI = 1.001–1.005 (*P* = 0.028)	151
Longer duration of psychosis	(45)	OLA/RIS	58.4	*P* = 0.027	77
	(57)	CLO/OLA^*^	42.2	OR = 1.053, 95% CI = 1.009–1.099 (*P* = 0.018)	116
Tobacco smoking	(87)	CLO/OLA/ RIS^*^	38.3	*P* = 0.047	120
	(73)	CLO/OLA/RIS/ QUE/PAL	41	*P* < 0.001	237
	(76)	OLA/RIS/QUE	49.6	OR = 0.6, 95% CI = 0.37–1.00 (*P* = 0.049)	476
HTR2C rs1414334 C-allele	(69)	CLO/OLA/RIS/ARI/QUE/	35	OR, 4.09, 95% CI, 1.41–11.89(*P* = 0.015)	162
	(93)	CLO/OLA/RIS^*^	25	OR = 4.09, 95% CI = 1.41–11.89 (*P* = 0.01)	112

*Patients with cumulative exposure to antipsychotics ≤ 2 weeks were categorized as the Minimal Antipsychotic Exposed Group, and the remainder were classified as the Antipsychotic Exposed Group*.

*Moderate risk group*.

* *Also other AP used in this study*.

*Age >40 years risk factor for MetS*.

*The main oral drug treatments were clozapine (n = 21), olanzapine (n = 31) and risperidone (n = 16); depot medication was received by 27 patients. No further details on AP that were being used*.

Various studies reported BMI to be a significant risk factor for MetS. Higher baseline BMI was found to be associated with MetS (85). Medved et al. (83), Saatcioglu et al. (57), and Hagg et al. (95), reported higher current BMI to be significantly correlating with MetS in their subjects (57, 83, 95). A BMI >25 was found to be associated with MetS by Grover et al. (71), while Lin et al. (61) and Yang et al. (55), reported this association for BMI >24. Higher antipsychotic dose was reported to be associated with MetS in two studies (43, 89). Duration of psychosis was found to be a risk factor for the development of MetS (45, 57). Three studies reported a significantly higher prevalence of MetS in tobacco smoking patients (73, 76, 87). One genetic factor, carriership of the variant rs1414334 C-allele, was found to increase the prevalence of MetS (69, 93).

## Discussion

In this systematic review that examines the clinical, biochemical and/ or genetic factors associated with MetS in patients using SGAs, we found male and female gender, higher age (at initiation of treatment), concomitant use of mood stabilizers, higher baseline BMI, higher current BMI, higher dose and longer duration of treatment to be positively associated with MetS in patients treated with clozapine. In studies with pooled antipsychotics, hypo-adiponectinemia, elevated CRP (≥ 3 mg/L), higher WBC count, female and male gender, older age, higher baseline BMI, higher current BMI, BMI >24, higher dose, longer duration of psychosis, tobacco smoking and HTR2C polymorphism were found to be positively associated with MetS. Overall, higher age, higher baseline BMI, higher current BMI and male as well as female gender were the only factors associated with MetS across all antipsychotics.

A large meta-analysis by Pillinger et al. (24), found male gender to predict greater vulnerability to antipsychotic-induced metabolic dysregulation. In this systematic review gender was also found to be a factor associated with MetS in patients treated with clozapine and in the pooled antipsychotics group. However, the results on this factor are not unequivocal since some studies found male gender while other studies found female gender to be associated with MetS. One possible explanation for these contradictory findings is related to age. In the general population, the risk of MetS increases with age in a gender-specific manner: under 50 years of age the risk is slightly higher in men, while over the age of 50 the risk is higher in women (97). In addition to gender, age was found to be a risk factor in the clozapine studies, as well as in the pooled groups. Given the fact that with older age the risk of MetS in the general population increases, this is not surprising. Due to variations in MetS prevalence between different countries, we expected to find an association between ethnicity and MetS. However, no clear association was found. It cannot be excluded that this incongruence is compounded by socio-economic factors.

Another factor associated with the development of MetS in studies including clozapine and SGAs was higher antipsychotic dosage. It has been suggested that not all SGA have the same propensity to induce metabolic disturbances, whereby clozapine and olanzapine appear to have concentration-dependent metabolic effects (98). Interestingly, drugs with high affinity for the H_1_-histamine, muscarinic, and α-adrenergic receptors, also seem to exhibit the strongest off-target metabolic effects, especially with higher doses (98).

As expected, considering the clinical role of CRP as cardiovascular risk indicator, elevated CRP (≥3 mg/L) was found to be positively associated with MetS. The finding of higher WBC, another inflammatory marker, is consistent with previous literature, however, the underlying mechanism explaining this association remains unclear (80). Furthermore, the association between concomitant use of mood stabilizers and MetS in patients treated with clozapine is in line with previous studies showing a higher MetS prevalence in patients receiving polypharmacy vs. monotherapy and studies reporting weight gain and MetS following treatment with mood stabilizers (10, 99–101). Our findings regarding hypoadiponectinemia support results from previous studies (102). Adiponectinemia is thought to have a normalizing effect on metabolic dysregulations (103).

Literature suggests that nicotine can reduce food intake and body weight (104) and might therefore reduce the risk of MetS (46, 74). Interestingly, in our systematic review, tobacco smoking was found to be positively associated with MetS. Tobacco smoking is known to cause upregulation of CYP1A1, CYP1A2, CYP2E1 and UGT, enzymes that are known to be involved in, among others, antipsychotic metabolism (105, 106). Smoking patients therefore would have lower plasma concentrations when comparing to non-smoking patients with the same dose. Nevertheless, physicians might prescribe these patients higher doses in order to reach the same plasma level, which could explain our finding (54, 89, 100).

Previous studies evaluating genetic factors observed inconsistent results on whether certain polymorphisms increase risk of MetS (Supplementary Tables 10, 11). To our knowledge, only the rs1414334 C allele of the HTR2C gene has been associated with MetS in multiple studies, although in pooled groups of antipsychotics. Although the mechanism of action is unclear, Mulder et al. (107), reported this polymorphism also to be significantly related to an increased risk of obesity in psychiatric patients treated with antipsychotics (107). This strengthens the idea that this polymorphism could be relevant.

One of the constituents of MetS is waist circumference (WC). A normal waist circumference differs for specific ethnic groups due to differences in cardiometabolic risk. For example, the relationship between WC and risk factors is such that men and women of South Asian descent present with a more severe metabolic risk profile than those of European descent at the same WC (108). As ethnic descent influences the relationship between WC and metabolic risk factors, current WC data derived from studies in European populations cannot be directly extrapolated to Asians. Furthermore, Asians have increased cardiometabolic risk with lower waist circumferences than other populations (109). Therefore, ethnic background should be considered when using WC as a marker of cardiovascular risk.

Naturally, several limitations may have influenced the results and interpretation of this systematic review. First, the included literature largely consisted of cross-sectional studies. Due to the nature of these studies, no definite conclusions regarding potential causal relationships may be made. Second, the antipsychotic agents used prior to the treatment drug were not recorded in most cross-sectional studies. If these antipsychotics included atypical antipsychotics, the associated factors could not be attributed to the SGA alone. For example, olanzapine prior to the initiation of clozapine leads to adverse metabolic consequences impacting weight, glucose and cholesterol (110). Besides, especially in the pooled groups of antipsychotics, the variation between SGA with their differing propensities to cause metabolic disturbances might have skewed the results. Third, a relatively large part of the studies was conducted in Asian countries. The results of these studies may therefore not be directly extrapolated to the European patient population since there are important metabolic differences partially related to lifestyle and diet between Asian and Caucasian patients on SGA (24). Twenty-seven studies were performed in Asian countries. Furthermore, a substantial number of the studies (40%) was performed in low- or middle-income countries (2 and 21, respectively), which may influence the generalizability of the results. More importantly, the inclusion of studies investigating different ethnicities is a problem regarding the genetic factors. The Chinese population can differ widely in genetic makeup compared to Europeans. This will have clinical implications for genotyping patients based on genetic findings that may not be relevant to the clinical population of interest. Fourth, several factors found to be associated with MetS (see Supplementary Tables 4–11) were only reported in a single study and thus excluded, while other factors were consistently identified in multiple studies. The results of the current study should therefore be interpreted with caution. Finally, we only included studies that used MetS as outcome measure to compare studies adequately. During the suitability screening in our literature search, we noticed that the majority of the initially identified studies investigated the effect of antipsychotics on one or more separate metabolic disturbances, instead of the MetS definition using ATP III, ATP III-A or IDF criteria. For example, the large meta-analysis by Pillinger et al. (24) investigated the effects of various antipsychotics on individual metabolic abnormalities, but not the syndrome as a whole. Therefore, these studies fell outside the scope of this systematic review. Potentially, this influenced the validity of this systematic review, leading to ambiguous and incomplete results.

Despite these limitations, the findings of this study are a promising first step toward the application of using clinical, biochemical and genetic information in personalized medicine. Evidence shows that MetS is highly prevalent among schizophrenia patients. Possibly, several factors interact to increase this risk in schizophrenia patients, including effects of SGAs, unhealthy lifestyle, and genetic and pathophysiological vulnerability. Further research is needed to elucidate how individual risk factors operate to increase this risk as well as how risk factors may interact to further increase MetS risk. Further research is also required to examine whether the contributions of these factors geographically differ. In this context, both clinical and preclinical studies may prove useful in the future to ascertain underlying pathophysiological mechanisms. Further risk factor management strategies are also required, involving pharmaceutical and nonpharmaceutical lifestyle interventions to try and counter the effects of such risk factors on MetS risk profiles in schizophrenia patients.

However, before applying these factors in clinical practice, by determining which patients have a high risk at developing MetS during SGA use, more research is required. Studies are needed using machine learning techniques to identify the exact molecular basis of the identified factors and to individually predict the risk to develop MetS in clinical practice. In this way, the discrepancy between life expectancy of patients with psychotic spectrum disorders and the general population may be reduced.

## Data Availability Statement

The original contributions presented in the study are included in the article/Supplementary Material, further inquiries can be directed to the corresponding author/s.

## Author Contributions

MS and SE performed the literature search. All authors contributed to the writing of the systematic review and read and approved the final manuscript.

## Conflict of Interest

The authors declare that the research was conducted in the absence of any commercial or financial relationships that could be construed as a potential conflict of interest.
