# Supplementary material for: Clinical, Biochemical and Genetic Variables Associated With Metabolic Syndrome in Patients With Schizophrenia Spectrum Disorders Using Second-Generation Antipsychotics: A Systematic Review
Source: Front Psychiatry. 2021 Mar 29;12:625935. doi: 10.3389/fpsyt.2021.625935 (PMC8044798; doi:10.3389/fpsyt.2021.625935)
Supplement: Supplementary file 1 [file Data_Sheet_1.docx]

Supplementary Material

Supplementary Results (Table 1-11) p.2

Search Strings p.29

PRISMA Checklist p.31

# 1. Supplementary Results

## Demographics

**Table 1:** Summary of study characteristics of the studies which identified factors in clozapine treatment

| **Study** | **Country** | **Study design** | **N** | **Male/**  **female (%)** | **Mean Age (years)** | **Mean duration of SGA treatment (years)** |
| --- | --- | --- | --- | --- | --- | --- |
| Lamberti *et al.*^1^ | VS | Cross-sectional study | 93 | 67/33 | 34.4 ± 8.7 | 5.9 ± 3.6 |
| Bai *et al.*^2^ | China | Retrospective cohort research | 188 | 64/36 | 43.3 ± 8.67 | 4.8 ± 2.3 |
| Ahmed *et al.*^3^ | Ireland | Cross-sectional study | 84 | 73/27 | 40.4 ± 10.6 | 1.57 ± 0.96 |
| Brunero *et al.*^4^ | Australia | Cross-sectional study | 73 | 62/38 | MetS group: 42 ±10.3  Non-MetS group: 35 ±8.4 (#2) | MetS group: 7.75 ± 4.77  Non-MetS group: 5.69 ± 4.89 (#2) |
| Josiassen *et al.*^5^ | VS | Cohort study | 25 | 64/36 | 45.8 ± 10.38 | 1-3 |
| Mulder *et al*.^6^ | Netherlands | Cross-sectional study | 78 | #1 | #1 | #6 Minimal 3 months |
| Steylen *et al.*^7^ | Netherlands | Cross-sectional study | 62 | 74/26 | 49.0 ± 14.2 | 9.2 ± 6 |
| Grover *et al.*^8^ | India | Cross-sectional study | 100 | 69/31 | 25.7 ± 11.6 | 4.65 ± 4.3 |
| Kang *et al*.^9^ | Korea | Cross-sectional study | 146 | 71/39 | 39.8 ± 8.5 | 4.3 ± 2.6 |
| Fernandez *et al.*^10^ | Venezuela | Case-control study | 56 | 88.6/21.4 | 39.1 ± 9.0 | #6 Minimal 3 months |
| Chen *et al*.^11^ | China | Cross-sectional study | 24 | #6 | MetSgroup: 46.4 ± 5  Non-MetSgroup: 44.4 ± 7.1 (#2) | #6 Minimal 1 year |
| Lee *et al.*^12^ | Korea | Retrospective cohort research | 113 | 63/37 | 36.0 ± 8.8 | 5.9 ± 3.4 |
| Zhang *et al*.^13^ | China | Cross-sectional study | 143 (#5) | #7 | 55.0 ± 7.4 | #6 Minimal 1 year |
| Zhang *et al.*^14^ | China | Cross-sectional study | 468 | 73/27 | 56.1 ± 7.4 | 5.9 ± 3.4 |
| Yang *et al.*^15^ | China | Case-control study & cross-sectional study | 621 (#5) | 57/43 | #2 MetSgroup: 44.42 ± 10.76  Non-MetSgroup: 44.16 ± 11.02 | #2 MetSgroup: 6.51 ± 3.84  Non-MetSgroup: 5.80 ± 4.31 |
| Popovic *et al.*^16^ | Serbia | Case-control study | 92 | 65/35 | #3 Men: 47.32 ± 8.69 Woman: 47.53 ±11.04 | #3 Men: 0.84 ± 0.28  Woman: 0.85 ± 0.17 |
| Yang *et al.*^17^ | China | Case–control study | 631 (#5) | #1 | #1 | #1 |
| Zhang *et al.*^18^ | China | Cross-sectional study | 576 (#5) | 77/23 | #2 MetSgroup: 57.1 ± 7.1  Non-MetSgroup: 55. 8 ± 8.5 | #2 MetSgroup: 17.48 ± 6.33  Non-MetSgroup: 17.25 ± 6.8 |
| Pinto *et al*.^19^ | Brazil | Cross-sectional study | 72 | #6 | 43 | #6 |
| Puangpetch *et al.*^20^ | Thailand | Cross-sectional study | 50 | 50/50 | #1 | #1 |
| Chen *et al*.^21^ | Taiwan | Cross-sectional study | 109 | 56/44 | 41.97 ± 9.26 | 19.03 ± 9.56 |

*** Partly also other (atypical) antipsychotics

| **Study** | **Country** | **Study design** | **N** | **Male/**  **female (%)** | **Mean Age (years)** | **Mean duration of SGA treatment (years)** |
| --- | --- | --- | --- | --- | --- | --- |
| Mulder *et al*.^6^ | Netherlands | Cross-sectional study | 71 | n/a | n/a | n/a |
| Kraemer *et al.*^22^ | Germany | Cross-sectional study | 476 | 49/51 | 45.2 ± 13.3 | n/a |
| Popovic *et al*.^16^ | Serbia | Case-control study | 93 | 76/24 | 48.13 ± 8.78 | 8.39 ± 2.15 |
| Popovic *et al*.^23^ | Serbia | Cross-sectional case-control study | 93 | 76/24 | 48.13 ± 8.78 | 8.39 ± 2.15 |
| Zhang *et al*.^24^ | China | Cross-sectional case-control study | 216 | 53/47 | 28.65 ± 3.52 | 35.09 ± 6.47 |
| Lu *et al*.^25^ | Taiwan | Cross-sectional study | 151 | 47/53 | 41.3 ± 12.1 | n/a |
| Japir *et al*.^26^ | Sudan | Cross-sectional study | 100 | 45/55 | n/a | n/a |

**Table 2:** Summary of study characteristics of the studies which identified factors in olanzapine and risperidone treatment

**Table 3:** Summary of study characteristics of the studies which identified factors in pooled SGA treatment

| **Study** | **Country** | **Study design** | **N** | **Male/**  **female (%)** | **Mean Age (years)** | **Mean duration of SGA treatment (years)** |
| --- | --- | --- | --- | --- | --- | --- |
| Kato *et al*.^27^ | United States | Cross-sectional study | 48 | 50/50 | 40.3 ± 12.0 | n/a |
| Hägg *et al*.^28^ | Sweden | Cross-sectional study | 269 | 66/34 | 46 ± n/a | 8.7 ± n/a |
| Mulder *et al*. ^29^ | Netherlands | Cross-sectional study | 112 | 66/34 | 36 ± 10 | n/a |
| Boke *et al*.^30^ | Turkey | Cross-sectional study | 231 | 75/25 | 38.5 ± 10.5 | n/a |
| Ellingrod *et al*.^31^ | United States | Cross-sectional study | 58 | 66/34 | 36.4 ± 10.0 | n/a |
| Lee *et al*.^32^ | China | Cross-sectional study | 75 | 46.7/53.3 | 34.8 ± 11.1 | 37.0 ± 21.5 |
| Ojala *et al*. ^33^ | Finland | Cross-sectional study | 221 | 89/11 | 41 | n/a |
| Yevtushenko *et al*.^34^ | United Kingdom | Cross-sectional study | 134 | 65/35 | 41.6 ± 11.8 | n/a |
| Bai *et al*.^35^ | Taiwan | Retrospective cohort study | 567 | 59/41 | 45.7 ± 27.6 | 3.8± 2.3 |
| Medved *et al.*^36^ | Croatia | Cohort study | 94 | 0/100 | 31.07 ± 7.86 | n/a |
| Patel *et al*.^37^ | United States | Randomised double-blind trial | 400 | 73/27 | 24.53 ± 5.8 | n/a |
| Fan *et al*.^38^ | United States | Cross-sectional study | 199 | 54/46 | 44 ± 11 | n/a |
| Van Winkel *et al*.^39^ | Netherlands | Cross-sectional study | 518 | 66/34 | 35.0 ± 11.1 | n/a |
| Kraemer *et al*.^22^ | Germany | Cross-sectional study | 476 | 49/51 | 45.2 ± 13.3 | n/a |
| Kuzman *et al*.^40^ | Croatia | Cohort study | 101 | 0/100 | 33.5 ± 10.6 | n/a |
| Lee *et al.*^41^ | Korea | Cross-sectional study | 145 | 51/49 | 35.8 ± 9.3 | 8.3 ± 6.2 |
| Ellingrod *et al*.^42^ | United States | Cross-sectional study | 237 | 51/49 | 44.7 ± 11.7 | n/a |
| Grover *et al*.^43^ | India | Cross-sectional study | 227 | 53.7/56.3 | 34.7 ± 12.8 | n/a |
| Liou *et al*.^44^ | Taiwan | Cross-sectional study | 456 | 66/34 | 48.4 ± 12.5 | 3.8 ± 2.3 |
| Risselada *et al*.^45^ | Netherlands | Cross-sectional study | 186 | 68/32 | 37 ± 11 | n/a |
| Liou *et al*.^46^ | Taiwan | Cross-sectional study | 456 | 66/34 | 48.4 ± 12.5 | 3.8 ± 2.3 |
| Lott *et al*.^47^ | United States | Cross-sectional study | 85 | 65/35 | 45.4 ± 11.7 | n/a |
| Miller *et al*.^48^ | United States | Cross-sectional study | 59 | 59/41 | 39.3 ± 11.4 | n/a |
| Roffeei *et al*.^49^ | Malaysia | Cross-sectional study | 206 | 67/33 | 39.9 ± 11.3 | n/a |
| Lin *et al*.^50^ | China | Cross-sectional cohort study | 329 | 56/44 | 47.48 ± 9.08 | n/a |
| Saatsioglu *et al*.^51^ | Turkey | Cross-sectional study |  | 42.2/57.8 | 37.4 ± 10.5 | 13.14 ± 9.04 |
| Yang *et al.*^52^ | China | Cross-sectional study | 357 | 56/44 | 47.96 ± 9.12 | n/a |
| Kraal *et al*.^53^ | United States | Cross-sectional study | 287 | 59.6/40.4 | Male: 44.16±11.34  Female: 46.89±11.34 | n/a |
| Chen *et al*.^54^ | Taiwan | Cross-sectional study | 262 | 55/45 | Male 43.5 ± 10.7  Female: 42.1 ± 10.3 | Male: 16.1 ± 8.8  Female: 15.0 ± 8.4 |
| Larsen *et al*.^55^ | Denmark | Cross-sectional study | 145 | 59.3/40.7 | 42.1 ± 10.6 | n/a |
| Chen *et al.*^21^ | Taiwan | Cross-sectional study | 157 | 48/52 | 41.1 ± 9.2 | 17.7±9.5 |
| Dehelean *et al*.^56^ | Romania | Cross-sectional cohort study | 77 | 53.2/46.8 | 42.48 ± 11.34 | n/a |
| Iruretagoyena *et al*.^57^ | Chile | Cross-sectional study | 148 | 77.7/22.3 | 21.06 ± 2.01 | 2.17 ± 2.15 |
| Ventriglio *et al*.^58^ | Italy | Cross-sectional study | 151 | 52.9/47.1 | 42.1 ± 12.4 | n/a |
| Bai *et al*. ^59^ |  |  |  |  |  |  |

**1.2 Quality assessment**

**Study participation**

There was a high risk of bias regarding study participation in twenty-one studies, seventeen had an unclear risk of bias and twenty a low risk of bias regarding study participation. This was because of one or two of the following problems: generalizability, inclusion and exclusion criteria were not adequately described, potential selection bias, no key characteristics list, only inpatients, small sample size or period of recruitment was not adequately described. See fig. 2 for the quality assessment of the included studies.

**Study attrition**

Four studies had a high risk of bias regarding study attrition, three had an unclear risk. The other fifty-one studies had a low risk of bias. The available study data on patients who dropped out did not adequately represent the study sample. The relationship between the prognostic factor and MetS is likely to be different for completing and non-completing participants in these studies. Three studies had an uncertain risk of bias with regards to study attrition.

**Prognostic factor measurement**

There was a low risk of bias regarding prognostic factor measurement in most of the studies. This means that the factors were adequately measured in all study participants; there was limited potential of bias. Three studies presented prognostic factor measurements that have a risk of bias, due to missing data, suboptimal metabolic measurements and analyses and use of subjective objective assessment tools. Two studies had an unclear risk of bias.

**Outcome measurement**

There was a low risk of bias regarding outcome measurements in almost all studies. MetS was clearly defined by using international standards and the measurements were valid and reliable for all studies.

**Study confounding**

The cross-sectional design of most of the included studies causes a high risk of confounding. Forty-five studies were classified as having a high risk of bias, seven had an unclear risk of bias and the other six had a low risk of bias. Factors such as sample size and subject heterogeneity could have confounded the observed results while not being accounted for in the study design or analysis. Studies in which extensive multivariate regressions were performed had a lower risk of bias.

**Statistical analysis and reporting**

There was a high risk of bias regarding statistical analysis and reporting in thirteen studies. The other fourty-five studies were classified as low risk, because the statistical analysis was appropriate for the design of the study and the potential for presentation of invalid or spurious results was limited.

**1.3 Clozapine**

Four studies investigated biochemical factors (see table 4). The retrospective cohort study of Lee *et al.* has shown that elevated levels of serum ALT were associated with MetS during clozapine treatment^12^. The retrospective cohort study of Bai *et al.* has shown that hypo-adiponectinemia was associated with MetS^2^. They found that the area under the receiver operating characteristic curve ± SD for adiponectin to predict MetS was 0.747 ± 0.026 (p < .001) and the cut-off value was 7.6 μg/mL, with 0.76 sensitivity and 0.63 specificity^2^. The cross-sectional study of Zhang *et al.*^14^ has shown that elevated levels of homocysteine were associated with MetS during clozapine treatment. Chen *et al.* ^21^ reported that orexin-A levels are a predictive variable of MS (adjusted OR = 0.04, 95% CI: 0.01–0.38 for the 2nd tertile and adjusted OR = 0.04, 95% CI 0.01–0.36 for the 3rd tertile, both values P < 0.01).

There are several factors for which disparate associations were found (see table 4). Three studies found female gender to be associated with MetS during clozapine use^5,7,14^, while three other studies found male gender to be associated with MetS during clozapine use^3,4,20^. Another factor which had inconsistent associations was age. Three studies found higher age to be related to MetS^7,14^; while two other studies did not find this association^7,14^. Also, clozapine duration, clozapine doses, diabetes in close family members and weight were variably associated with MetS during clozapine treatment. Studies which associated longer clozapine duration with MetS were: Lamberti *et al.*^1^ and Josiassen *et al.*^5^ while according to three other studies longer clozapine duration was not associated with MetS^1,5^. For higher clozapine doses two studies claimed it to be associated with MetS^4,5^, while two other studies found it not to be associated with MetS^7,14^. While two studies found diabetes in close family members not to be associated with MetS^3,7^, Popovic *et al.* found diabetes in close family members to be related to MetS^23^. Lastly, higher current weight was a factor with ambivalent research outcomes. According to Brunero *et al.* higher current weight was associated with MetS^4^, while according to Ahmed *et al.* it was not^3^.

Individual studies found ten genetic factors to be related to MetS during clozapine use (see table 4). These factors are: homozygous Met/Met genotype of BDNF gene^13^, SREBPF2 single-nucleotide polymorphisms (SNPs): rs1052717 and rs2267443^15^, T-allele of rs11654081 of SREBF1 gene^17^, TT homozygotes of rs11654081 of SREBF1 gene^17^, Pro/Ala genotype of the PPAR-γ2 gene^10^, C allele of rs1414334 of HTR2C gene^6^ and CC homozygosity of rs498177 of HTR2C gene in female patients^59^. Associations were not only found for genes, also RNA was associated with MetS. Levels of C3 mRNA^18^ were also associated with MetS. The levels of C3 mRNA expression in patients with MetS were higher than those without MetS. Differentially-expressed miRNAs (DERs): Hsa-miR-330-3p, has-miR-18a and has-miR-106b were also related to MetS among patients with psychotic spectrum disorders who use clozapine^11^.

Nine studies reported genetic factors not to be related to MetS during clozapine treatment (see table 5). These factors are: COMT SNPs: rs4633, rs4680 and rs4818^14^, C3 SNP: rs2277984^18^. C3 SNP rs2277984 was marginally associated with MetS. Other genetic factors which were found to be not related to MetS during clozapine use were: TaqIA,-141C and rs6280 polymorphisms of the ANKK1, DRD2 and DRD3 genes^19^, Pro/Pro genotype of the PPAR-γ2 gene^10^, polymorphisms of PPARγ 161C/T (rs3856806)^9^, PPAR*α* V227A (rs1800234)^9^, Val/Val and Val/Met genotype of BDNF gene^13^, several SNPs of the SREBF1 gene (rs11652861, rs7503334, rs6502618, rs13306738, rs1889018, rs4925118, rs2297508, rs8066560, rs7222480 and rs11868035) and SCAP gene (rs1078224, rs17079634, rs9683033, rs12487736 and rs2306628)^17^ and SREBPF2 gene (rs4822063, rs17002737, rs2267439, rs5996080, rs5996078, rs1569451, rs2228314 and rs17379759)^15^. Several SNPs in the promoter region of the HTR2C gene were also not associated with MetS^9^.

**Table 4**: Results regarding factors which were associated with MetS in clozapine

| **Factor** | **Study/studies** | **MetS prevalence (%)** | **Test statistics** | **N** |
| --- | --- | --- | --- | --- |
| Hypo-adiponectinemia | Bai *et al.*^2^ | 28.4 | OR = 0.783 (P < 0.001) | 188 |
| Elevated levels of serum ALT | Lee *et al.* ^12^ | 34.5 to 46.9 | Significant; no P value mentioned | 113 |
| Elevated levels of homocysteine | Zhang *et al.*^14^ | 43.2 | P < 0.01 | 468 |
| Lower levels of orexin-A | Chen *et al.*^21^ | 35.8 | P < 0.01 | 109 |
| Male gender | Ahmed *et al.*^3^ | 46,6 | OR = 11.18 (P = 0.013) | 84 |
|  | Brunero *et al.*^4^ | 61.6 | P = 0.009; | 73 |
|  | Puangpetch *et al.*^20^ | 36 | OR = 4.33 (P = 0.02) | 50 |
| Female gender | Josiassen *et al.*^5^ | 64 | P < 0.05 (F = 4.9) | 25 |
|  | Zhang *et al.*^14^ | 43.2 | P = 0.04; | 468 |
|  | Steylen *et al.*^7^ | 61 | P = 0.012 | 62 |
| (Higher) insulin levels | Ahmed *et al.* ^3^ | 46.6 | OR = 1.10 (P = 0.039) | 84 |
| Concomitant use of mood stabilizers | Bai *et al.* ^2^ | 28.4 | OR =2.642 (P = 0.041) | 188 |
|  | Steylen *et al.*^7^ | 61 | P = 0.023 | 62 |
| (Higher) age at initiation of clozapine treatment | Bai *et al.*^2^ | 28.4 | OR = 1.056 (P = 0.049) | 188 |
|  | Josiassen *et al.* ^5^ | 64 | Statistical trend | 25 |
| (Higher) age | Bai *et al.*^2^ | 28.4 | P =0.009 | 188 |
|  | Brunero *et al.*^4^ | 61.6; | OR = 1.083 (#2) (P = 0.007) | 73 |
|  | Lamberti *et al.*^1^ | 53.8 | P < 0.001 | 93 |
| (Higher) baseline BMI | Bai *et al.* ^2^ | 28.4 | OR = 1.226 (P < 0.001) | 188 |
|  | Josiassen *et al.*^5^ | 64 | F = 16.12 (P < 0.005) | 25 |
| (Higher) current BMI | Ahmed *et al.* ^8^ | 46.6 | OR = 1.38 (P = 0.001) | 84 |
|  | Grover *et al.*^8^ | 47; | P = 0.001 | 100 |
|  | Lamberti *et al.*^1^ | 53.8; | P < 0.0001 | 93 |
|  | Brunero *et al.* ^4^ | 61.6 | P = 0.001 | 73 |
| (Higher) current weight | Brunero *et al.* ^4^ | 61.6 | P = 0.001 | 73 |
| (Higher) baseline glucose | Josiassen *et al.* ^5^ | 64 | F = 4.1 (P < 0.05) | 25 |
| (Higher) triglycerides/high density lipoprotein ratio | Brunero *et al.*^4^ | 61.6 | P = 0.001 | 73 |
| (longer) education in years | Grover *et al.*^8^ | 47 | P =0.013 | 100 |
| (Higher) clozapine dose | Josiassen *et al.*^5^ | 64 | Statistical trend | 25 |
|  | Brunero *et al.*^4^ | 61.6 | P = 0.03 | 73 |
| (Longer) clozapine duration | Josiassen *et al.*^5^ | 64 | F=5.97 (P < 0.01) | 25 |
|  | Lamberti *et al.* ^1^ | 53.8 | Statistical trend (P = 0.06) | 93 |
| Use of antidepressants | Lee *et al.*^12^ | 34.5 - 46.9 | Significant, no P-value mentioned | 113 |
| Diabetes in close family members | Popovic *et al.*^23^ | 41.3 | OR = 14.127, 95% CI = 2.407 – 82892 (P = 0.003) | 285 |
| Pro/Ala genotype of the PPAR-γ2 gene | Fernandez *et al.* ^10^ | #5 | P = 0.003 | 56 |
| C allele of rs1414334 of HTR2C gene | Mulder *et al*. ^6^ | 34.6 | OR = 9.2, 95% CI = 1.95 - 43.45 | 78 |
| CC homozygotes of rs498177 of HTR2C gene in female patients | Bai *et al*.^59^ | 21.7 | P = 0.008 | 157* |
| SREBPF2 SNPs: rs1052717 | Yang *et al.*^15^ | 41.8 | OR= 1.54, 95% CI = 1.18–2.01  (P = 0.002) | 621 |
| SREBPF2 SNPs: rs2267443 | Yang *et al.*^15^ | 41.8 | OR= 1.34, 95% CI = 1.04–1.72  (P = 0.003) | 621 |
| T-allele of rs11654081 of SREBF1 gene | Yang *et al.*^17^ | 41.3 | OR= 2.34, 95% CI = 1.3 1-4.14  (P = 0.033) | 631 |
| TT homozygotes of rs11654081 of SREBF1 gene | Yang *et al.*^17^ | 41.3 | OR = 2.32, 95% CI = 1.3–4.11  (P = 0.026) | 631 |
| Homozygous Met/Met genotype of BDNF gene | Zhang *et al*.^13^ | 39.9 | OR = 2.39, 95% CI = 1.05–5.41  (P = 0.039) | 143 |
| Levels of C3 mRNA | Zhang *et al.*^18^ | 42.7 | P = 0.02 | 576 |
| DERs: Hsa-miR-330-3p | Chen *et al*.^11^ | - | P = 0.00934 | 24 |
| DERs: has-miR-18a | Chen *et al*.^11^ | - | P = 0.001686 | 24 |
| DERs: has-miR-106b | Chen *et al*.^11^ | - | P = 0.007531 | 24 |

***** Female patients

**Table 5**: Results regarding genetic factors which were **not** associated with MetS in clozapine

| **Factor** | **Study** | **MetS prevalence (%)** | **Test statistics** | **N** |
| --- | --- | --- | --- | --- |
| TaqIA, -141C and rs6280 polymorphisms of the ANKK1gene | Pinto *et al*. ^19^ | 47.2 | P > 0.05 | 72 |
| TaqIA, -141C and rs6280 polymorphisms of DRD2 gene | Pinto *et al.*^19^ | 47.2 | P > 0.05 | 72 |
| TaqIA, -141C and rs6280 polymorphisms of DRD3 gene | Pinto *et al*.^19^ | 47.2 | P > 0.05 | 72 |
| COMT SNPs: rs4633, rs4680, and rs4818 | Zhang *et al.*^14^ | 43.2 | P > 0.05 | 468 |
| C3 SNP: rs2277984 | Zhang *et al.*^18^ | 42.7 | OR = 1.36, 95% CI = 1.07 – 1.72  (P = 0.06) | 576 |
| Pro/Pro genotype of the PPAR-γ2 gene | Fernandez *et al.*^10^ | #5 | P > 0.05 | 56 |
| Polymorphisms of PPARγ 161C/T (rs3856806) | Kang *et al*. ^9^ | 47.3 | P = 0.996 | 146 |
| Polymorphisms of PPAR*α* V227A (rs1800234) | Kang *et al*.^9^ | 47.3 | P = 0.539 | 146 |
| Polymorphism in the HTR2C gene (rs518147 -697 G/C) | Kang *et al*. ^9^ | 47.3 | P = 0.229 | 146 |
| Polymorphism in the HTR2C gene (rs3813928 759C/T) | Kang *et al*. ^9^ | 47.3 | P = 0.212 | 146 |
| SREBPF2 gene (rs4822063, rs17002737, rs2267439, rs5996080, rs5996078, rs1569451, rs2228314 and rs17379759) | Yang *et al.*^15^ | 41.8 | P > 0.05 | 621 |
| SREBF1 gene (rs11652861, rs7503334, rs6502618, rs13306738, rs1889018, rs4925118, rs2297508, rs8066560, rs7222480 and rs11868035) and SCAP gene (rs1078224, rs17079634, rs9683033, rs12487736 and rs2306628) | Yang *et al.*^17^ | 41.3 | P > 0.05 | 631 |
| Val/Val and Val/Met genotype of BDNF gene***** | Zhang *et al*.^13^ | 39.9 | P > 0.05 | 143 |

**1.4 Olanzapine**

Eight factors were found to be associated with MetS in patients treated with MetS (see table 6). Five factors were found not to be associated with MetS (see table 7). Popovic *et al.* studied risk factors associated with the use of olanzapine ^16^. They found that enhanced C-reactive protein (CRP) levels, over the cutoff point of 5mg/L, were significantly associated with MetS during olanzapine treatment. Zhang *et al*. measured the plasma brain-derived neurotrophic factor (BDNF) levels in schizophrenia patients with or without MetS and observed a close relationship between MetS and reduced levels of BDNF^24^. Plasma tumor necrosis factor-alpha (TNF-alfa) levels, however, were significantly lower in patients with MetS. Microalbuminuria (>300 mg/L) was found not to be a predictor for MetS ^23^.

Five clinical factors were found to be related to MetS in patients treated with olanzapine (see table 6). Two studies reported male gender to be related with MetS during olanzapine treatment^23,41^. However, another study found gender not to be related with MetS during olanzapine treatment^26^. In a cross-sectional case-control study, Popovic *et al*. found diabetes mellitus type 2 in close family members, hyperlipidemia in close family members and BMI to be statistically significant parameters, as strong predictors for MetS in their patient sample ^23^. With regards to dose and duration of treatment, Japir *et al.* found no specific association between the dose of olanzapine or the duration of treatment as long as the dose of olanzapine is 5 mg or more for a duration of nine months or more^26^.

Only one study was found that investigated genetic factors in olanzapine treatment in isolation. Mulder *et al*. reported that polymorphisms in the promoter region of the HTR2C C-allele was not a significant predictor in patients treated with olanzapine ^16^.

**Table 6**: Results regarding factors which were associated with MetS in olanzapine

| **Factor** | **Study/studies** | **MetS prevalence (%)** | **Test statistics** | **N** |
| --- | --- | --- | --- | --- |
| C-reactive protein above the cutoff point of 5mg/L | Popovic *et al.* ^16^ | 34.4 | P = 0.042 | 93 |
| Lower plasma brain-derived neurotrophic factor (BDNF) levels | Zhang *et al*.^24^ | 44 | P = 0.012 | 216 |
| Higher plasma tumor necrosis factor-alpha (TNF-alpha) | Zhang *et al*.^24^ | 44 | P = 0.026 | 216 |
| Male gender | Lee *et al.*^41^ | 39 | OR = 4.18, 95% CI = 1.93 – 9.03 | 41 |
|  | Popovic *et al*. ^23^ | 34.4 | P = 0.049 | 93 |
| Diabetes in close family members | Popovic *et al*. ^16^ | 34.4 | P = 0.039 | 93 |
| Hyperlipidemia in close family | Popovic *et al.* ^16^ | 34.4 | P = 0.008 | 93 |
| BMI | Popovic *et al.* ^16^ | 34.4 | P = 0.002 | 93 |
| Previous olanzapine treatment vs. none | Kraemer *et al.* ^22^ | 48.4 | P = 0.0071 | 62 |

**Table 7**: Results regarding factors which were **not** associated with MetS in olanzapine.

| **Factor** | **Study/studies** | **MetS prevalence (%)** | **Test statistics** | **N** |
| --- | --- | --- | --- | --- |
| Gender | Japir *et al*.^26^ | 45 | P = 0.646 | 100 |
| Dose | Japir *et al*.^26^ | 45 | P = 0.56 | 100 |
| Duration of treatment | Japir *et al*.^26^ | 45 | P = 0.43 | 100 |
| Microalbuminuria (>300 mg/L) | Popovic *et al.* ^23^ | 34.4 | P = 0.999 | 93 |
| Polymorphisms in the promoter region of the HTR2C gene rs3813929 and rs518147 | Mulder *et al*. ^6^ | 23.9 | OR = 2.63, 95% CI = 0.50–13.74 (P > 0.05) | 71 |

**1.5 Risperidone**

Only one study that investigated genetic factors in risperidone treatment isolated was identified. Mulder *et al*. found that polymorphisms in the promoter region of the HTR2C C-allele was a significant predictor^6^. The variant rs1414334 C allele was strongly associated with the metabolic syndrome in patients using risperidone.

**Table 8:** Results regarding factors which were associated with MetS in risperidone

| **Factor** | **Study/studies** | **MetS prevalence (%)** | **Test statistics** | **N** |
| --- | --- | --- | --- | --- |
| C allele of rs1414334 of HTR2C gene | Mulder *et al*.^6^ | 22 | OR = 5.35, 95% CI = 1.26 – 22.83 (P < 0.05) | 63 |

**1.6 Pooled results**

The majority of the studies that investigated potential associations between biochemical, clinical or genetic factors and MetS were analyzed in groups rather than in isolation, mostly due to sample size constraints. Table 9 shows the results for studies that investigated biochemical, clinical and genetic factors that were found to be associated with MetS in patients treated with SGA. Table 10 shows the factors that were found not to be related to MetS in patients using SGA.

Six studies investigated biochemical factors and their association with MetS in patients treated with (atypical) antipsychotics. In a Taiwanese cohort study, Bai *et al*. performed a cross-sectional multiple logistic regression and found hypo-adiponectinemia to be a significant factor in predicting the development of MetS in patients treated with either clozapine, olanzapine or risperidone^35^. Similar results were reported by Chen *et al*.^54^. They also found a significant association between increased leptin levels and the occurrence of MetS. In another Taiwanese study, it was reported that orexin-A levels were a predictive variable for MetS, following results indicating that higher levels were significantly associated with a lower risk of MetS ^21^. Their patients were receiving clozapine, aripiprazole, haloperidol, amisulpride, and ziprasidone. Kraemer *et al*., showed in an observational study that a CRP level of ≥3 mg/L was significantly associated with MetS compared to normal values^22^. Patients in this study were receiving olanzapine, risperidone, quetiapine, amisulpride, aripiprazole, clozapine, ziprasidone, paliperidone or a combination of these. A small proportion of patients (3.4%) received typical antipsychotics. Miller *et al*. also found a higher CRP level to be associated with MetS in a cross-sectional study with patients receiving risperidone, ziprasidone, aripiprazole, quetiapine, haloperidol, clozapine, olanzapine, paliperidone, perphenazine mostly as monotreatment^48^. Some patients received two types of antipsychotics. Apart from increased CRP-levels, differential and total white blood cell (WBC) counts were a significant predictor of MetS. Patients with MetS had significantly higher total WBC counts, monocytes and CRP-levels. The relation between WBC and MetS was also found by Fan *et al*. in their cross-sectional study in patients receiving olanzapine, risperidone, and typical antipsychotics (mostly haloperidol and fluphenazine)^38^. No significant differences between these groups in WBC values were found. Lin *et al*. demonstrated in a cross-sectional cohort study that abnormal non-HDL-C levels were a predictor for MetS in male patients receiving clozapine, risperidone, ziprasidone, quetiapine, aripiprazole, zotepine, amisulpride, or olanzapine^50^.

Twenty-three clinical factors were found to be related to MetS (table 9). Previous studies evaluating the influence of gender on MetS, observed inconsistent results on whether gender is a predictor for MetS. Three studies found male gender to be associated with MetS. Lee *et al*. performed a retrospective chart review in patients receiving a monotherapy with aripiprazole, olanzapine or risperidone^21^. A logistic regression analysis showed that sex significantly predicted the presence of MetS when corrected for independent variables of age, sex, type of current antipsychotic, dose as well as duration of treatment and illness. Chen *et al*. also found male gender significantly associated with MetS in their sample with patients receiving clozapine, aripiprazole, haloperidol, amisulpride, and ziprasidone^21^. Kraemer *et al*. also found this relation between male gender and MetS in an observational study with patients receiving olanzapine, risperidone, quetiapine, amisulpride, aripiprazole, clozapine, ziprasidone, paliperidone or a combination of these^22^. Interestingly, these outcomes with regards to gender are contradictory to several other studies, which report female gender to be associated with MetS. Lee *et al.*, Grover *et al*., and Boke *et al*. reported female gender to be a predictor of MetS in their patient samples^30,32,53^. A close to significant (P = 0.05) association between female gender and MetS in patients treated with SGA including clozapine, olanzapine, quetiapine, risperidone, paliperidone, iloperidone was demonstrated by Kraal *et al*. ^43^. Surprisingly, several other studies reported gender not to be a risk factor for MetS ^35^.

Several studies investigated the influence of age on MetS in patients using SGA. Nine studies reported a significantly higher MetS prevalence in higher aged patients ^21,30,34,35,41,42,52,58^. Grover *et al*., showed age to be a risk factor especially in patients with age > 35^43^.

Six studies identified BMI as a significant predictor of MetS. Bai *et al*. performed retrospective reviews of the study subjects' medical records and showed that BMI at initiation of antipsychotic treatment and BMI increase after initiation of antipsychotic treatment were significantly associated with MetS^35^. Yang *et al*. reported more precisely a BMI >24 kg/m^2^ to be the strongest risk factor for MetS^52^. Grover *et al*. found similar results for higher BMI and BMI >25^43^. Medved *et al*. showed that only BMI was a significant predictor of MetS in their patient sample^36^. Higher current BMI was also found to be associated with MetS by Saatcioglu *et al*.,^51^ and Hägg *et al*.^28.^ Finally, Lin *et al*. scrutinized the factor BMI further and investigated the influence of gender. It appears that BMI was a strong risk factor for MetS with a slightly higher ratio in men (men: OR=6.092; women: OR=5.886)^50^.

Patel *et al*. analyzed data from the CAFE study (randomized double-blind trial) and concluded that treatment emergent MetS occurred in a significantly lower proportion of African American patients than in patients of other ethnicities (8.5% vs. 17.2% respectively)^27^. To determine the effect of Hispanic vs. non-Hispanic patients, Kato *et al*. investigated the prevalence of MetS in their patients^32,58^. It was found that Hispanics had a significantly higher prevalence of metabolic syndrome at 74% than non-Hispanics at 41%. Interestingly, Roffeei *et al.* found no significant differences between Malaysian, Chinese, and Indian ethnic groups^56^.

Patel *et al*., performed an exploratory analysis to investigate whether previous SGA-exposure predisposes patients to MetS^21^. It was found that treatment emergent MetS was reported significantly fewer in minimally antipsychotic exposed patients than in previously antipsychotic exposed patients (8.6% vs. 16.2%). Treatment-emergent MetS was reported in a large number of patients at the end of the study. Surprisingly, a cross-sectional study by Chen *et al*. found no significant association between the duration of antipsychotic treatment in years and MetS ^34^.

Several studies showed that the occurrence of MetS is dose related. Higher dosage (CPZ-eq doses) of SGA was identified as being a risk factor that enhances the likelihood of developing MetS^32,58^. Subsequently, in the latter study, it was also found that risk of MetS was greater among Schizoaffective disorder patients than Schizophrenia subjects and among people with a higher age^58^.

In a double-centered observational study by Dehelean *et al*. the metabolic profile of 77 Schizoaffective and Schizophrenia subjects using olanzapine or risperidone was analyzed^56^. Patients presenting MetS had a longer duration of psychosis and longer duration of long-acting injectable (LAI) treatment than those without MetS. Interestingly, the presence of the metabolic syndrome, were not influenced by the type of the antipsychotic or by the associated mood stabilizer.

Tobacco smoking was found to be significantly related to MetS in three studies. Ellingrod *et al*. reported MetS prevalence to be highly associated with smoking status^42^, just as Kraemer *et al*.^22^ and Yevtushenko *et al*.^34^. However, Chen *et al*., Lee *et al*., and Saatcioglu *et al.* did not find an association between smoking status and MetS prevalence^21,32,51^.

Concomitant non-psychiatric medication was found to be associated with MetS^22^. In a study performed by Larsen *et al*., it was reported that MetS was significantly more pronounced in schizophrenia patients with vs. without prediabetes (76.2% vs. 40.9%)^55^. Ojala *et al*. identified the risk of MetS to be twice as high for non-alcoholics and for patients not using SSRIs in their patient population^33^. In another study, Grover *et al*. found a significant correlation between MetS and urban locality, and between MetS and being employed^43^. Finally, Ventriglio *et al*. found no significant association between MetS and orally administered vs. long-acting injected (LAI) antipsychotics^58^.

**Table 9:** Results regarding factors which were associated with MetS in pooled groups of SGAs

| **Factor** | **Study/studies** | **Antipsychotics** | **MetS prevalence (%)** | **Test statistics** | **N** |
| --- | --- | --- | --- | --- | --- |
| Hypo-adiponectinemia | Bai *et al*.^35^ | CLO/OLA/RIS | 23.8 | P < 0.0001 | 567 |
|  | Chen *et al*.^54^ | CLO/OLA | 33.2 | OR = 0.882, 95% CI = 0.841–0.925 (P = 0.005) | 262 |
| Leptin | Chen *et al*. ^54^ | CLO/OLA | 33.2 | OR = 1.045, 95% CI = 1.013–1.077 (P < 0.001) | 262 |
| Lower levels of orexin-A | Chen *et al.*^21^ | CLO/ARI/HAL/AMI | 31.2 | OR = 0.04, 95% CI = 0.01 – 0.38 (P < 0.01) | 157 |
| CRP ≥ 3 mg/L | Kraemer *et al*. ^22^ | CLO/OLA/RIS | 49.6 | P = 0.0062 | 476 |
|  | Miller *et al.*^48^ | CLO/OLA/RIS/  ARI/QUE/  PAL/HAL/ZIP*** | 32.2 | P = 0.04 | 59 |
| abnormal non-HDL-C: ≥ 130 mg/dL in male patients | Lin *et al*.^50^ | CLO/OLA/RIS/ARI/  QUE/ZOT/AMI/ZIP | 23.7 | P < 0.001 | 329 |
| Higher total WBC count | Miller *et al*.^48^ | CLO/OLA/RIS/ARI/  QUE/PAL/HAL/ZIP*** | 32.2 | P = 0.001 | 59 |
|  | Fan *et al*.^38^ | OLA/RIS*** | 53.8 | P = 0.004 | 199 |
| Monocytes | Miller *et al*.^48^ | CLO/OLA/RIS/ARI/  QUE/PAL/HAL/ZIP*** | 32.2 | P = 0.02 | 59 |
| Higher HbA1C | Hägg *et al*.^28^ | CLO/OLA** | 34.6 | P = 0.002 | 269 |
| Higher fasting serum insulin | Hägg *et al*.^28^ | CLO/OLA** | 34.6 | P < 0.001 | 269 |
| Male gender | Lee *et al.*^41^ | OLA/RISP/ARI | 31.7 | OR = 2.09, 95% CI = 1.49 – 2.70  (P < 0.05) | 145 |
|  | Chen *et al*. ^21^ | CLO/ARI/HAL/AMI/ZIP | 31.2 | OR = 1.45, 95% CI = 0.60 –3.48 (P < 0.05) | 157 |
|  | Kraemer *et al*.^22^ | OLA/RIS/QUE | 49.6 | P = 0.0185 | 476 |
| Female gender | Lee *et al*.^32^ | OLA/RISP | 14.7 | P < 0.01 | 75 |
|  | Kraal *et al*.^53^ | QUE/RISP/  ILO/PAL** | 41.1 | P = 0.05 | 112 |
|  | Boke *et al*.^30^ | N/A | 32.0 | P = 0.005 | 231 |
|  | Grover *et al*. ^43^ | CLO/OLA/RIS/  QUE | 43.6 | OR = 1.81, 95% CI = 1.07 – 3.08 (P = 0.027) | 227 |
| (Higher) age | Lee *et al.*^41^ | OLA/RISP/ARI | 31.7 | P = 0.02 | 145 |
|  | Ventriglio *et al*.^58^ | CLO/OLA/RIS/ARI/  QUE/PAL/HAL | 31.8 | P = 0.029 | 151 |
|  | Chen *et al.* ^21^ | CLO/ARI/HAL/  AMI/ZIP | 31.2 | OR = 1.03, 95% CI = 0.98 – 1.09 (P < 0.05) | 157 |
|  | Ellingrod *et al*.^42^ | CLO/OLA/RIS/  QUE/PAL | 41 | P < 0.001 | 237 |
|  | Bai *et al*.^35^ | CLO/OLA/RIS | 23.8 | P = 0.007 | 567 |
|  | Yang *et al*.^52^ | OLA/RIS**** | 37.8 | P = 0.012 | 357 |
|  | Boke *et al*.^30^ | N/A | 32.0 | P = 0.026 | 231 |
|  | Yevtushenko *et al*.^34^ | CLO/OLA/RIS****** | 38.3 | P = 0.003 | 120 |
| Age > 35 | Grover *et al*. ^43^ | CLO/OLA/RIS/QUE | 43.6 | OR = 3.37, 95% CI = 1.94 – 5.86 (P < 0.001) | 227 |
| (Higher) baseline BMI | Bai *et al*.^35^ | CLO/OLA/RIS | 23.8 | P = 0.007 | 567 |
| (Higher) current BMI | Medved *et al.*^36^ | OLA/RISP | 27 | P < 0.001 | 40 |
|  | Saatcioglu *et al*.^51^ | CLO/OLA **** | 42.2 | P = 0.018 | 116 |
|  | Hägg *et al*.^28^ | CLO/OLA**** | 34.6 | P < 0.001 | 269 |
| (Higher) BMI increase after initiation of antipsychotic treatment | Bai *et al*.^35^ | CLO/OLA/RIS | 23.8 | P = 0.007 | 567 |
| BMI > 25 | Grover *et al*.^43^ | CLO/OLA/RIS/  QUE | 43.6 | P < 0.001 | 227 |
| BMI > 24 | Lin *et al*.^50^ | CLO/OLA/RIS/  ARI/QUE/  ZOT/AMI/ZIP | 23.7 | P < 0.001 | 329 |
|  | Yang *et al*.^52^ | OLA/RIS**** | 37.8 | P < 0.001***** | 357 |
| Earlier AAP exposure* | Patel *et al*.^37^ | OLA/RISP/QUE | 18.6 | P < 0.05 | 307 |
| Ethnicities other than African American | Patel *et al*.^37^ | OLA/RISP/QUE | 18.6 | P < 0.05 | 307 |
| Hispanic vs. non-Hispanic | Kato *et al*.^27^ | CLO/OLA/RISP/  HAL | 63 | P = 0.024 | 48 |
| (Higher) dose | Lee *et al*.^32^ | OLA/RISP | 14.7 | P < 0.01 | 75 |
|  | Ventriglio *et al*.^58^ | CLO/OLA/RIS/  ARI/QUE/  PAL/HAL | 31.8 | P = 0.028 | 151 |
| Schizoaffective disorder vs. schizophrenia | Ventriglio *et al*.^58^ | CLO/OLA/RIS/  ARI/QUE/  PAL/HAL | 31.8 | P = 0.035 | 151 |
| Longer duration of psychosis | Dehelean *et al*. ^56^ | OLA/RIS | 58.4 | P = 0.027 | 77 |
|  | Saatcioglu *et al*.^51^ | CLO/OLA **** | 42.2 | P = 0.018 | 116 |
| Longer duration of treatment | Dehelean *et al*. ^56^ | OLA/RIS | 58.4 | P < 0.0001 | 77 |
| Tobacco smoking | Yevtushenko *et al.*^34^ | CLO/OLA/  RIS****** | 38.3 | P = 0.047 | 120 |
|  | Ellingrod *et al*.^42^ | CLO/OLA/RIS/  QUE/PAL | 41 | P < 0.001 | 237 |
|  | Kraemer *et al*. ^22^ | OLA/RIS/QUE | 49.6 | P = 0.049 | 476 |
| Receiving non-psychiatric concomitant medication | Kraemer *et al*. ^22^ | OLA/RIS/QUE | 49.6 | P = 0.059 | 476 |
| Prediabetes | Larsen *et al*.^55^ | CLO/OLA | 65.5 | P < 0.05 | 145 |
| Non-alcoholism | Ojala *et al*.^33^ | CLO/OLA/RISP/  ARI/QUE/SUL/  SER/ZOT | 32 | P = 0.039 | 221 |
| Non-SSRI use | Ojala *et al*. ^33^ | CLO/OLA/RISP  ARI/QUE/SUL/  SER/ZOT | 32% | P = 0.024 | 221 |
| Being employed | Grover *et al*. ^43^ | CLO/OLA/RIS/  QUE | 43.6 | OR = 2.12, 95% CI = 1.09–4.10 (P = 0.025) | 227 |
| Urban locality | Grover *et al*. ^43^ | CLO/OLA/RIS/  QUE | 43.6 | OR = 2.08, 95% CI = 1.15–3.75 (P = 0.014) | 227 |
| Leptin genotype -2548AG/GG | Yevtushenko *et al.*^34^ | CLO/OLA/RISP | 44.0 | P = 0.032 | 98 |
| Leptin genotype -2548AG/GG in -759CC 5HT2C patients | Yevtushenko *et al.*^34^ | CLO/OLA/RISP | 47.1 | P = 0.003 | 70 |
| INSIG2 rs11123469-C homozygous genotype | Liou *et al*.^44^ | CLO/OLA/RISP | 22.8 | P = 0.001 | 456 |
| C-C-C haplotype of rs11123469-rs10185316- rs1559509 of the INSIG2 gene | Liou *et al*.^44^ | CLO/OLA/RISP | 22.8 | P = 0.0023 | 456 |
| MTHFR A1298C/C genotype | Van Winkel *et al*.^39^ | CLO/OLA | 33.2 | P = 0.003 | 236 |
| MTHFR C677T allele vs. C-allele | Ellingrod *et al*.^31^ | CLO/OLA/RIS/  ARI/QUE/HAL/ZIP | 40 | P = 0.02 | 58 |
| MTHFR rs1801133 T-allele | Roffeei *et al*.^49^ | OLA/RIS/PAL*** | 59.7 | P = 0.016 | 206 |
| MTHFR 677T and COMT 158Val alleles | Ellingrod *et al*.^31^ | CLO/OLA/RIS/  QUE/PAL | 41 | P < 0.001 | 237 |
| HTR2C:c.1 – 142948(GT)_n_ 13 repeats allele | Mulder *et al,*^29^ | CLO/OLA/RIS****** | 36 | OR = 3.12, 95% CI, 1.13 – 8.16  (P < 0.05) | 45 |
| HTR2C polymorphisms  rs518147 (–697) C | Mulder *et al,* ^29^ | CLO/OLA/RIS****** | 25 | (OR = 2.62, 95% CI, 1.00–6.85  (P = 0.049) | 112 |
| HTR2C polymorphisms  rs1414334 C | Mulder *et al,* ^29^ | CLO/OLA/RIS****** | 25 | OR = 4.09, 95% CI, 1.41–11.89  (P = 0.01) | 112 |
|  | Risselada *et al*.^45^ | CLO/OLA/RIS/  ARI/QUE/ | 35 | P = 0.015 | 162 |
| Combined genotype with carriers of the HTR2C:c.1 – 142948(GT)_n_ 13 repeat allele, the common allele rs3813929 ( – 759) C, and the variant alleles rs518147 (–697) C and rs1414334 C | Mulder *et al*.^29^ | CLO/OLA/RIS****** | 25 | OR = 4.69, 95% CI = 1.34–16.45 (P > 0.05) | 112 |
| TT homozygotes of rs11654081 of SREBF1 gene | Yang *et al.*^52^ | CLO/OLA/RIS | 40.3 | P = 0.026** | 722 |
| FTO rs9939609 A-allele | Roffeei *et al*.^49^ | OLA/RIS/PAL*** | 59.7 | P = 0.047 | 206 |
| LEPR rs1137101  GG/G allele | Roffeei *et al*.^49^ | OLA/RIS/PAL*** | 59.7 | P = 0.022 | 206 |
| COMT G/G allele promotor region methylation**** | Lott *et al*.^47^ | CLO/OLA/RIS/QUE/PAL | 36 | P = 0.002 (site 1); P = 0.001 (site 2) | 85 |
| MTTP polymorphism rs1800591 (-493G/T) | Liou *et al*.^46^ | CLO/OLA/RIS | 22.8 | P = 0.0003 | 456 |

* Patients with cumulative exposure to antipsychotics ≤2 weeks were categorized as the Minimal Antipsychotic Exposed Group, and the remainder were classified as the Antipsychotic Exposed Group.

** Moderate risk group

**** Also other AP used in this study

***** Age >40 years risk factor for MetS

****** The main oral drug treatments were clozapine (n=21), olanzapine (n=31) and risperidone (n=16); depot medication was received by 27 patients. No further details on AP that were being used.

**Table 10**: Results regarding factors which were **not** associated with MetS in patients taking SGA.

| **Factor** | **Study** | **SGA** | **MetS prevalence (%)** | **Test statistics** | **N** |
| --- | --- | --- | --- | --- | --- |
| Gender | Yevtushenko *et al.*^34^ | CLO/OLA/RIS | 38 | P > 0.05 | 120 |
|  | Patel *et al*.^37^ | OLA/RIS/QUE | 18.6 | P > 0.05 | 307 |
|  | Ojala *et al*.^33^ | CLO/OLA/RIS/  ARI/QUE/SUL/  SER/ZOT | 32 | OR = 0.79, 95% CI 0.26 – 2.37 (P > 0.05) | 221 |
|  | Kraal *et al*.^53^ | CLO/OLA | 64.6 | P = 0.12 | 79 |
|  | Dehelean *et al.*^56^ | OLA/RIS | 58.4 | P > 0.05 | 77 |
|  | Iruretagoyena *et al.*^57^ | CLO/OLA | 44.7 | P = 0.271 | 148 |
|  | Roffeei *et al*.^49^ | CLO/OLA/RIS/  ARI/QUE/SUL/  PAL/HAL/AMI*** | 59.7 | P > 0.05 | 206 |
|  | Lin *et al*.^50^ | CLO/OLA/  RIS/ARI/QUE/  ZOT/AMI/ZIP | 23.7 | P = 0.387 | 329 |
| (Higher) age | Ojala *et al*.^33^ | CLO/OLA/RISP/  ARI/QUE/SUL/  SER/ZOT | 32 | OR = 1.00, 95% CI 0.97–1.03 (P > 0.05) | 221 |
|  | Dehelean *et al.*^56^ | OLA/RIS | 58.4 | P > 0.05 | 77 |
| (Longer) duration of AAP treatment | Chen *et al*.^21^ | CLO/ARI/  HAL/AMI/  ZIP | 31.2 | OR = 1.00, 95% CI = 0.95 – 1.05 (P < 0.05) | 157 |
| (Longer) duration of illness | Lin *et al*.^50^ | CLO/OLA/  RIS/ARI/QUE/  ZOT/AMI/ZIP | 23.7 | P = 0.207 | 329 |
| Ethnicity | Roffeei *et al*.^49^ | CLO/OLA/RIS/  ARI/QUE/SUL/  PAL/HAL/AMI*** | 59.7 | P > 0.05 | 206 |
| Tobacco smoking history | Chen *et al*.^21^ | CLO/ARI/  HAL/AMI/  ZIP | 31.2 | OR = 1.82, 95% CI = 0.69–4.80 (P > 0.05) | 157 |
|  | Lee *et al*.^32^ | OLA/RIS | 14.7 | P = 0.056 | 75 |
|  | Saatcioglu *et al*.^51^ | CLO/OLA | 42.2 | OR = 2.102, 95% CI = 0.994 – 4.448 (P = 0.052) | 116 |
| Orally vs. Injected antipsychotics | Ventriglio *et al*.^58^ | CLO/OLA/RIS/  ARI/QUE/  PAL/HAL | 31.8 | P = 0.98 | 151 |
| 5 HT2C receptor genotype -759C/CC or 759T/CT | Yevtushenko *et al.*^34^ | CLO/OLA/RISP | 39.0 | P = 0.97 | 118 |
|  | Kuzman *et al*.^40^ * | OLA/RIS | 31.7 | P = 0.615 | 101 |
| MTHFR C677T polymorphism | Van Winkel *et al*.^49^ | CLO/OLA | 33.2 | P = 0.121 | 236 |
| MTHFR 1298A/C polymorphism | Ellingrod *et al*. ^49^ | CLO/OLA/RIS/ARI/QUE/HAL/ZIP | 40******** | P > 0.05 | 58 |
| MDR1 exon 21 G2677T polymorphism | Kuzman *et al*. ^40^ * | OLA/RIS | 31.7 | P = 0.423 | 101 |
| MDR1 exon 26 C3435T polymorphism | Kuzman *et al*.^40^ * | OLA/RIS | 31.7 | P = 0.225 | 101 |
| SREBF1 gene (rs11652861, rs7503334, rs6502618, rs13306738, rs1889018, rs4925118, rs2297508, rs8066560, rs7222480 and rs11868035) and SCAP gene (rs1078224, rs17079634, rs9683033, rs12487736 and rs2306628) | Yang *et al.*^17^ | CLO/OLA/RIS | 40.3 | P > 0.05 | 722 |
| ADIPOQ (rs182052, rs7649121) | Roffeei *et al*. ^49^ | OLA/RIS/PAL*** | 59.7 | P > 0.05 | 206 |
| ADRA2A (rs1800544) | Roffeei *et al*. ^29^ | OLA/RIS/PAL*** | 59.7 | P > 0.05 | 206 |
| BDNF (rs6265) | Roffeei *et al*. ^49^ | OLA/RIS/PAL*** | 59.7 | P > 0.05 | 206 |
| DRD2 (rs1079598) | Roffeei *et al*.^49^ | OLA/RIS/PAL*** | 59.7 | P > 0.05 | 206 |
| HTR2A (rs6311) | Roffeei *et al.*^49^ | OLA/RIS/PAL*** | 59.7 | P > 0.05 | 206 |
| Polymorphism in HTR2C (rs3813929) | Roffeei *et al*.^49^ | OLA/RIS/PAL*** | 59.7 | P > 0.05 | 206 |
|  | Risselada *et al*.^45^ | CLO/OLA/RIS/  QUE/ARI | 35 | P > 0.05 | 162 |
| Polymorphism in HTR2C (rs3813929 T) | Mulder *et al*., ^6^ | CLO/OLA/RIS** | 31.7 | OR = 0.70, 95% CI = 0.29 – 1.68 (P > 0.05) | 41 |
|  | Mulder *et al*., ^49^******* | CLO/OLA/RIS****** | 31 | OR = 0.94, 95% CI = 0.49 –1.81 (P > 0.05) | 67 |
| Polymorphism in HTR2C (rs3813928 A / rs3813929 T) | Mulder *et al*.^6^ | CLO/OLA/RIS** | 27 | OR = 1.18, 95% CI = 0.40 – 3.47 (P > 0.05) | 26 |
| Polymorphisms in the promoter region of the HTR2C gene (HTR2C:c.1-142948(GT)_n_) | Mulder *et al*.^6^ ******* | CLO/OLA/RIS****** | 35.8 | OR = 1.69, 95% CI = 0.75 – 3.81 | 67 |
|  | Mulder *et al.*^49^ ******* | CLO/OLA/RIS****** | 38 | OR = 2.09, 95% CI = 1.12 – 3.91 | 112 |
| HTR2C (rs518147) | Roffeei *et al*. ^49^ | OLA/RIS/PAL*** | 59.7 | P > 0.05 | 206 |
| HTR2C (rs518147 C) | Mulder *et al*. ^47^ | CLO/OLA/RIS****** | 34.2 | P > 0.05 | 73 |
|  | Mulder *et al.*^49^ ******* | CLO/OLA/RIS****** | 36 | P > 0.05 | 121 |
| LEP (rs7799039) | Roffeei *et al*. ^47^ | OLA/RIS/PAL*** | 59.7 | P > 0.05 | 206 |
| MC4R (rs8087522) | Roffeei *et al*. ^49^ | OLA/RIS/PAL*** | 59.7 | P > 0.05 | 206 |
| MTHFR (rs1801131) | Roffeei *et al*.^49^ | OLA/RIS/  PAL*** | 59.7 | P > 0.05 | 206 |
| PMCH (rs11111201, rs7973796) | Roffeei *et al*.^49^ | OLA/RIS/  PAL*** | 59.7 | P > 0.05 | 206 |
| COMT A/A allele promotor region methylation**** | Lott *et al*.^47^ | CLO/OLA/RIS/QUE/PAL | 36 | P = 0.1895 (site 1); P = 0.468 (site 2) | 85 |

* Only female patients

** Other antipsychotics used were bromperidol (n = 2), flupentixol (n = 2), haloperidol (n = 2), perphenazine (n = 1), pimozide (n = 1), quetiapine (n = 1), and zuclopenthixol (n = 2). Polypharmacy was present in 16 (14%) of the included patients.

## *** Olanzapine, risperidone, paliperidone were used in more than 10% of study participants. Other AAP (clozapine, sulpiride, amisulpride, aripirazole, quetiapine, haloperidol, chlorpromazine, perphenazine, trifluoperazine and fluanxol) were used only by small proportion of participants.

**** Others: typical antipsychotics (n = 17) or a combination of antipsychotics (n = 29).

***** Only in male patients

****** and other typical antipsychotics

******* Pooled analysis of patients from Mulder *et al*.^6^ and Mulder *et al*.^49^

******** MetS prevalence in entire group

**2. Search strings**

**Search string Pubmed**:

Domain:

#1 Search (“Psychotic Disorder”[Title/Abstract] OR (“Psychotic Disorders”[Title/Abstract] OR Psychosis[Title/Abstract] OR Psychoses[Title/Abstract] OR Schizophrenia[Title/Abstract] OR “Schizophreniform disorder”[Title/Abstract] OR “Schizophreniform disorders”[Title/Abstract] OR “Schizoaffective disorder”[Title/Abstract] OR “Schizoaffective disorders”[Title/Abstract] OR “Psychotic affective disorder”[Title/Abstract] OR “Psychotic affective disorders”[Title/Abstract] OR “Delusional Disorder”[Title/Abstract] OR “Delusional Disorders”[Title/Abstract] OR “Delusional Parasitosis”[Title/Abstract] OR “Short-term psychotic disorder “OR “Short-term psychotic disorders “OR Catatonia [Title/Abstract] OR “Paranoid Disorder”[Title/Abstract] OR “Paranoid Disorders”[Title/Abstract] OR “psychotic patient”[Title/Abstract] OR “psychotic patients”[Title/Abstract])
– **154065 hits**

Outcome**:**#2 = Search (“Metabolic syndrome”[Title/Abstract] OR “Metabolic syndromes”[Title/Abstract] OR “insulin resistance syndrome”[Title/Abstract] OR “insulin resistance syndromes”[Title/Abstract] OR “Metabolic X Syndrome”[Title/Abstract] OR “Metabolic X Syndromes”[Title/Abstract] OR “Syndrome X”[Title/Abstract] OR “X syndrome”[Title/Abstract] OR “Metabolic Cardiovascular Syndrome”[Title/Abstract] OR “Metabolic Cardiovascular Syndromes”[Title/Abstract] OR “Plurimetabolic Syndrome”[Title/Abstract] OR “Plurimetabolic Syndromes”[Title/Abstract] OR “Dysmetabolic Syndrome”[Title/Abstract] OR “Dysmetabolic Syndromes”[Title/Abstract] OR “lipid abnormality”[Title/Abstract] OR “lipid abnormalities”[Title/Abstract] OR “waist circumference”[Title/Abstract] OR “waist size”[Title/Abstract] OR “waist sizes”[Title/Abstract] OR triglyceride[Title/Abstract] OR triglycerides[Title/Abstract] OR HDL[Title/Abstract] OR “cholesterol level”[Title/Abstract] OR “cholesterol levels”[Title/Abstract] OR “blood pressure”[Title/Abstract] OR “blood pressures”[Title/Abstract] OR “fasting glucose”[Title/Abstract] OR “fasting glucoses”[Title/Abstract])
**-** 511650 hits

Determinant:

#3: Search (“Clozapine”[Title/Abstract] OR “Leponex”[Title/Abstract] OR “Olanzapine”[Title/Abstract] OR “Zyprexa”[Title/Abstract] OR “Risperidone”[Title/Abstract] OR “Risperdal”[Title/Abstract] OR “Quetiapine”[Title/Abstract] OR “Aripiprazole”[Title/Abstract] OR “Abilify”[Title/Abstract] OR “Ziprasidone”[Title/Abstract] OR “Geodon”[Title/Abstract] OR “Lurasidone”[Title/Abstract] OR “Latuda”[Title/Abstract] OR “Paliperidone”[Title/Abstract] OR “Asenapine”[Title/Abstract] OR “Sertindole”[Title/Abstract] OR “Iloperidone”[Title/Abstract] OR “Fanapt”[Title/Abstract] OR “Sulpiride”[Title/Abstract] OR “Brexpiprazole”[Title/Abstract] OR “Rexulti”[Title/Abstract] OR “Cariprazine”[Title/Abstract] OR “Vraylar”[Title/Abstract] OR “Reagila”[Title/Abstract])
– 37639 hits

#1 AND #2 AND #3 🡪 697 hits

**Search string Embase:**
(‘Psychotic Disorder’:ti,ab,kw OR ‘Psychotic Disorders’:ti,ab,kw OR Psychosis:ti,ab,kw OR Psychoses:ti,ab,kw OR Schizophrenia:ti,ab,kw OR ‘Schizophreniform disorder’:ti,ab,kw OR ‘Schizophreniform disorders’:ti,ab,kw OR ‘Schizoaffective disorder’:ti,ab,kw OR ‘Schizoaffective disorders’:ti,ab,kw OR ‘Psychotic affective disorder’:ti,ab,kw OR ‘Psychotic affective disorders’:ti,ab,kw OR ‘Delusional Disorder’:ti,ab,kw OR ‘Delusional Disorders’:ti,ab,kw OR ‘Delusional Parasitosis’:ti,ab,kw OR ‘Short-term psychotic disorder‘:ti,ab,kw OR ‘Short-term psychotic disorders‘:ti,ab,kw OR Catatonia:ti,ab,kw OR ‘Paranoid Disorder’:ti,ab,kw OR ‘Paranoid Disorders’:ti,ab,kw OR ‘psychotic patient’:ti,ab,kw OR ‘psychotic patients’:ti,ab,kw) AND (‘Metabolic syndrome’:ti,ab,kw OR ‘Metabolic syndromes’:ti,ab,kw OR ‘insulin resistance syndrome’:ti,ab,kw OR ‘insulin resistance syndromes’:ti,ab,kw OR ‘Metabolic X Syndrome’:ti,ab,kw OR ‘Metabolic X Syndromes’:ti,ab,kw OR ‘Syndrome X’:ti,ab,kw OR ‘X syndrome’:ti,ab,kw OR ‘Metabolic Cardiovascular Syndrome*’:ti,ab,kw OR ‘Plurimetabolic Syndrome*’:ti,ab,kw OR ‘Dysmetabolic Syndrome’:ti,ab,kw OR ‘Dysmetabolic Syndromes’:ti,ab,kw OR ‘lipid abnormality’:ti,ab,kw OR ‘lipid abnormalities’:ti,ab,kw OR ‘waist circumference’:ti,ab,kw OR ‘waist circumferences’:ti,ab,kw OR ‘waist size’:ti,ab,kw OR ‘waist sizes’:ti,ab,kw OR triglyceride:ti,ab,kw OR triglycerides:ti,ab,kw OR HDL:ti,ab,kw OR ‘cholesterol level’:ti,ab,kw OR ‘cholesterol levels’:ti,ab,kw OR ‘blood pressure’:ti,ab,kw OR ‘blood pressures’:ti,ab,kw OR ‘fasting glucose’:ti,ab,kw OR ‘fasting glucoses’:ti,ab,kw) AND (“Clozapine”:ti,ab,kw OR “Leponex”:ti,ab,kw OR “Olanzapine”:ti,ab,kw OR “Zyprexa”:ti,ab,kw OR “Risperidone”:ti,ab,kw OR “Risperdal”:ti,ab,kw OR “Quetiapine”:ti,ab,kw OR “Seroquel”:ti,ab,kw OR “Aripiprazole”:ti,ab,kw OR “Abilify”:ti,ab,kw OR “Ziprasidone”:ti,ab,kw OR “Geodon”:ti,ab,kw OR “Lurasidone”:ti,ab,kw OR “Latuda”:ti,ab,kw OR “Paliperidone”:ti,ab,kw OR “Asenapine”:ti,ab,kw OR “Sertindole”:ti,ab,kw OR “Iloperidone”:ti,ab,kw OR “Fanapt”:ti,ab,kw OR “Sulpiride”:ti,ab,kw OR “Brexpiprazole”:ti,ab,kw OR “Rexulti”:ti,ab,kw OR “Cariprazine”:ti,ab,kw OR “Vraylar”:ti,ab,kw OR “Reagila”:ti,ab,kw)

**🡪 1356 hits**

Search results from both Pubmed (697 results) and EMBASE (1356 results) resulted in 2053 articles. The snowball method resulted in an additional 8 articles. After removing duplicates, 683 unique articles were screened for suitability for inclusion, utilizing the search criteria defined above, by reading the titles and abstracts. Fig. 1 of the main text provides additional information on the search and yield.

**PRISMA Checklist**

| **Section/topic** | **#** | **Checklist item** | **Reported on page #** |
| --- | --- | --- | --- |
| **TITLE** | | |  |
| Title | 1 | Identify the report as a systematic review, meta-analysis, or both. | 1 |
| **ABSTRACT** | | |  |
| Structured summary | 2 | Provide a structured summary including, as applicable: background; objectives; data sources; study eligibility criteria, participants, and interventions; study appraisal and synthesis methods; results; limitations; conclusions and implications of key findings; systematic review registration number. | 2 |
| **INTRODUCTION** | | |  |
| Rationale | 3 | Describe the rationale for the review in the context of what is already known. | 3 – 4 |
| Objectives | 4 | Provide an explicit statement of questions being addressed with reference to participants, interventions, comparisons, outcomes, and study design (PICOS). | 4 |
| **METHODS** | | |  |
| Protocol and registration | 5 | Indicate if a review protocol exists, if and where it can be accessed (e.g., Web address), and, if available, provide registration information including registration number. | n/a |
| Eligibility criteria | 6 | Specify study characteristics (e.g., PICOS, length of follow-up) and report characteristics (e.g., years considered, language, publication status) used as criteria for eligibility, giving rationale. | 5 |
| Information sources | 7 | Describe all information sources (e.g., databases with dates of coverage, contact with study authors to identify additional studies) in the search and date last searched. | 5 |
| Search | 8 | Present full electronic search strategy for at least one database, including any limits used, such that it could be repeated. | p. 28 – 29 (suppl.) |
| Study selection | 9 | State the process for selecting studies (i.e., screening, eligibility, included in systematic review, and, if applicable, included in the meta-analysis). | 5 |
| Data collection process | 10 | Describe method of data extraction from reports (e.g., piloted forms, independently, in duplicate) and any processes for obtaining and confirming data from investigators. | 5 |
| Data items | 11 | List and define all variables for which data were sought (e.g., PICOS, funding sources) and any assumptions and simplifications made. | 5 |
| Risk of bias in individual studies | 12 | Describe methods used for assessing risk of bias of individual studies (including specification of whether this was done at the study or outcome level), and how this information is to be used in any data synthesis. | 6 |
| Summary measures | 13 | State the principal summary measures (e.g., risk ratio, difference in means). | 5 |
| Synthesis of results | 14 | Describe the methods of handling data and combining results of studies, if done, including measures of consistency (e.g., I^2^) for each meta-analysis. | 5 |

| **Section/topic** | **#** | **Checklist item** | **Reported on page #** |
| --- | --- | --- | --- |
| Risk of bias across studies | 15 | Specify any assessment of risk of bias that may affect the cumulative evidence (e.g., publication bias, selective reporting within studies). | 13 - 14 |
| Additional analyses | 16 | Describe methods of additional analyses (e.g., sensitivity or subgroup analyses, meta-regression), if done, indicating which were pre-specified. | n/a |
| **RESULTS** | | |  |
| Study selection | 17 | Give numbers of studies screened, assessed for eligibility, and included in the review, with reasons for exclusions at each stage, ideally with a flow diagram. | 7 |
| Study characteristics | 18 | For each study, present characteristics for which data were extracted (e.g., study size, PICOS, follow-up period) and provide the citations. | 2 – 6 (suppl.) |
| Risk of bias within studies | 19 | Present data on risk of bias of each study and, if available, any outcome level assessment (see item 12). | 13 – 14 |
| Results of individual studies | 20 | For all outcomes considered (benefits or harms), present, for each study: (a) simple summary data for each intervention group (b) effect estimates and confidence intervals, ideally with a forest plot. | 15 – 17;  10 – 26 (suppl.) |
| Synthesis of results | 21 | Present the main results of the review. If meta-analyses are done, include for each, confidence intervals and measures of consistency. | 8 |
| Risk of bias across studies | 22 | Present results of any assessment of risk of bias across studies (see Item 15). | 7 – 8 |
| Additional analysis | 23 | Give results of additional analyses, if done (e.g., sensitivity or subgroup analyses, meta-regression [see Item 16]). | n/a |
| **DISCUSSION** | | |  |
| Summary of evidence | 24 | Summarize the main findings including the strength of evidence for each main outcome; consider their relevance to key groups (e.g., healthcare providers, users, and policy makers). | 9 – 10 |
| Limitations | 25 | Discuss limitations at study and outcome level (e.g., risk of bias), and at review-level (e.g., incomplete retrieval of identified research, reporting bias). | 10 – 11 |
| Conclusions | 26 | Provide a general interpretation of the results in the context of other evidence, and implications for future research. | 11 |
| **FUNDING** | | |  |
| Funding | 27 | Describe sources of funding for the systematic review and other support (e.g., supply of data); role of funders for the systematic review. | 11 |

Page 1 of 2

*From:*  Moher D, Liberati A, Tetzlaff J, Altman DG, The PRISMA Group (2009). Preferred Reporting Items for Systematic Reviews and Meta-Analyses: The PRISMA Statement. PLoS Med 6(7): e1000097. doi:10.1371/journal.pmed1000097

For more information, visit: **www.prisma-statement.org**.

Page 2 of 2

1.         Lamberti JS, Costea GO, Olson D, Crilly JF, Maharaj K, Tu X, et al. Diabetes mellitus among outpatients receiving clozapine: prevalence and clinical-demographic correlates. The Journal of Clinical Psychiatry [Internet]. 2005;66(7):900–6. Available from: http://www.ncbi.nlm.nih.gov/pubmed/16013906

2.         Bai YM, Chen J-Y, Yang W-S, Chi Y-C, Liou Y-J, Lin C-C, et al. Adiponectin as a potential biomarker for the metabolic syndrome in Chinese patients taking clozapine for schizophrenia. The Journal of Clinical Psychiatry [Internet]. 2007;68(12):1834–9. Available from: http://www.ncbi.nlm.nih.gov/pubmed/18162013

3.         Ahmed M, Hussain I, O’Brien SM, Dineen B, Griffin D, McDonald C. Prevalence and associations of the metabolic syndrome among patients prescribed clozapine. Irish Journal of Medical Science [Internet]. 2008;177(3):205–10. Available from: http://www.ncbi.nlm.nih.gov/pubmed/18461270

4.         Brunero S, Lamont S, Fairbrother G. Prevalence and Predictors of Metabolic Syndrome Among Patients Attending an Outpatient Clozapine Clinic in Australia. Archives of Psychiatric Nursing [Internet]. 2009 Oct 2;23(3):261–8. Available from: https://www.psychiatricnursing.org/article/S0883-9417(08)00137-4/abstract

5.         Josiassen R, Filmyer D, Curtis J, Shaughnessy R, Joseph A, Parson R, et al. An Archival, Follow-Forward Exploration of the Metabolic Syndrome in Randomly Selected, Clozapine-Treated Patients. Clinical Schizophrenia & Related Psychoses [Internet]. 2009 Oct 2;3(2):87–96. Available from: http://clinicalschizophrenia.org/doi/abs/.10.3371/CSRP.3.2.3

6.         Mulder H, Cohen D, Scheffer H, Gispen-de Wied C, Arends J, Wilmink FW, et al. HTR2C gene polymorphisms and the metabolic syndrome in patients with schizophrenia: a replication study. Journal of Clinical Psychopharmacology [Internet]. 2009;29(1):16–20. Available from: http://www.ncbi.nlm.nih.gov/pubmed/19142101

7.         Steylen PMJ, Heijden FMMA van der, Kok JDH, Tuinier S, Verhoeven WMA. Metabool syndroom bij de behandeling met clozapine. Pharmaceutisch Weekblad. 2009;144(3):96–100.

8.         Grover S, Nebhinani N, Chakrabarti S, Avasthi A, Kulhara P. Metabolic syndrome among patients receiving clozapine: A preliminary estimate. Indian Journal of Pharmacology [Internet]. 2011 Oct 2;43(5):591. Available from: http://www.ijp-online.com/article.asp?issn=0253-7613

9.         Kang SH, Lee J il, Chang AK, Joo YH, Kim CY, Kim SY. Genetic polymorphisms in the HTR2C and peroxisome proliferator-activated receptors are not associated with metabolic syndrome in patients with schizophrenia taking clozapine. Psychiatry Investigation [Internet]. 2011 Sep [cited 2020 Oct 4];8(3):262–8. Available from: /pmc/articles/PMC3182393/?report=abstract

10.        Fernández E, Carrizo E, Connell L, Baptista T. Pro12Ala polymorphism of the PPAR-γ2 gene, metabolic syndrome and response to metformin in clozapine-treated patients [Internet]. Vol. 137, Schizophrenia Research. Schizophr Res; 2012 [cited 2020 Oct 4]. p. 262–3. Available from: https://pubmed.ncbi.nlm.nih.gov/22377103/

11.        Chen J, Yang L, Liu D, Cui D, Yu S, Li Y, et al. MicroRNA Microarray Analysis Combined with Interaction Network Analysis to Investigate the Influence of Clozapine to Metabolic Syndrome. International Journal of Pharmacology [Internet]. 2013 Oct 2;9(6):366–72. Available from: http://www.scialert.net/abstract/?doi=ijp.2013.366.372

12.        Lee NY, Roh MS, Kim SH, Jung DC, Yu HY, Sung KH, et al. The prevalence of metabolic syndrome and its association with alanine  aminotransferase in clozapine-treated Korean patients with schizophrenia. International clinical psychopharmacology [Internet]. 2013;28(2):71–9. Available from: http://dx.doi.org/10.1097/YIC.0b013e32835b99bd

13.        Zhang Y, Chen M, Wu Z, Chen J, Yu S, Fang Y, et al. Association study of Val66Met polymorphism in brain-derived neurotrophic factor gene  with clozapine-induced metabolic syndrome: preliminary results. PloS one [Internet]. 2013;8(8):e72652. Available from: http://dx.doi.org/10.1371/journal.pone.0072652

14.        Zhang Y, Chen M, Chen J, Wu Z, Yu S, Fang Y, et al. Metabolic syndrome in patients taking clozapine: prevalence and influence of  catechol-O-methyltransferase genotype. Psychopharmacology. 2014;231(10):2211–8.

15.        Yang L, Chen J, Liu D, Yu S, Cong E, Li Y, et al. Association between SREBF2 gene polymorphisms and metabolic syndrome in  clozapine-treated patients with schizophrenia. Progress in neuro-psychopharmacology & biological psychiatry. 2015;56:136–41.

16.        Popovic I, Ravanic D, Djukic-Dejanovic S, Jankovic S, Popovic V. P.3.d.017 Prevalence and predictors of the metabolic syndrome in patients on the long term atypical antipsychotic treatment. European Neuropsychopharmacology [Internet]. 2015 Oct 2;25:S490. Available from: https://linkinghub.elsevier.com/retrieve/pii/S0924977X15306696

17.        Yang L, Chen J, Li Y, Wang Y, Liang S, Shi Y, et al. Association between SCAP and SREBF1 gene polymorphisms and metabolic syndrome in  schizophrenia patients treated with atypical antipsychotics. The world journal of biological psychiatry : the official journal of the World  Federation of Societies of Biological Psychiatry. 2016;17(6):467–74.

18.        Zhang C, Zhang Y, Cai J, Chen M, Song L. Complement 3 and metabolic syndrome induced by clozapine: a cross-sectional study  and retrospective cohort analysis. The pharmacogenomics journal. 2017;17(1):92–7.

19.        Pinto JAF, Freitas PHB de, Nunes FDD, Granjeiro PA, Santos LL dos, Machado RM. Prevalence of polymorphisms in the ANKK1, DRD2, DRD3 genes and metabolic syndrome in  refractory schizophrenia. Revista latino-americana de enfermagem. 2018;26:e2983.

20.        Puangpetch A, Unaharassamee W, Jiratjintana N, Koomdee N, Sukasem C. Genetic polymorphisms of HTR2C, LEP and LEPR on metabolic syndromes in patients treated with atypical antipsychotic drugs. The Journal of Pharmacy and Pharmacology [Internet]. 2018;70(4):536–42. Available from: http://www.ncbi.nlm.nih.gov/pubmed/29441581

21.        Chen P-Y, Chen C-H, Chang C-K, Kao C-F, Lu M-L, Lin S-K, et al. Orexin-A Levels in Relation to the Risk of Metabolic Syndrome in Patients with Schizophrenia Taking Antipsychotics. International Journal of Neuropsychopharmacology [Internet]. 2018 Oct 2;22(1):28–36. Available from: https://tmu.pure.elsevier.com/en/publications/orexin-a-levels-in-relation-to-the-risk-of-metabolic-syndrome-in-

22.        Kraemer S, Minarzyk A, Forst T, Kopf D, Hundemer HP. Prevalence of metabolic syndrome in patients with schizophrenia, and metabolic changes after 3 months of treatment with antipsychotics - results from a German observational study. BMC Psychiatry [Internet]. 2011 Nov 1 [cited 2020 Oct 4];11. Available from: https://pubmed.ncbi.nlm.nih.gov/22044502/

23.        Popović I, Ravanić D, Janković S, Milovanović D, Folić M, Stanojević A, et al. Long-Term Treatment with Olanzapine in Hospital Conditions: Prevalence and  Predictors of the Metabolic Syndrome. Srpski arhiv za celokupno lekarstvo. 2015;143(11–12):712–8.

24.        Zhang C, Fang X, Yao P, Mao Y, Cai J, Zhang Y, et al. Metabolic adverse effects of olanzapine on cognitive dysfunction: A possible  relationship between BDNF and TNF-alpha. Psychoneuroendocrinology. 2017;81:138–43.

25.        Lu M-L, Chen C-H, Kuo P-T, Lin C-H, Wu T-H. Application of plasma levels of olanzapine and N-desmethyl-olanzapine to monitor  metabolic parameters in patients with schizophrenia. Schizophrenia research. 2018;193:139–45.

26.        Mohamed Japir A, Osman AH. Olanzapine-Induced Metabolic Syndrome What Can We Learn from Africa, Sudan. Journal of Psychiatry. 2019;22(1).

27.        Kato MM, Currier MB, Gomez CM, Hall L, Gonzalez-Blanco M. Prevalence of Metabolic Syndrome in Hispanic and Non-Hispanic Patients With Schizophrenia. Primary Care Companion to The Journal of Clinical Psychiatry [Internet]. 2004 Oct 2;6(2):74–7. Available from: https://www.ncbi.nlm.nih.gov/pmc/articles/PMC427602/

28.        Hägg S, Lindblom Y, Mjörndal T, Adolfsson R. High prevalence of the metabolic syndrome among a Swedish cohort of patients with  schizophrenia. International clinical psychopharmacology. 2006;21(2):93–8.

29.        Mulder H, Franke B, van der-Beek van der AA, Arends J, Wilmink FW, Scheffer H, et al. The association between HTR2C gene polymorphisms and the metabolic syndrome in  patients with schizophrenia. Journal of clinical psychopharmacology. 2007;27(4):338–43.

30.        Boke O, Aker S, Sarisoy G, Saricicek EB, Sahin AR. Prevalence of Metabolic Syndrome among Inpatients with Schizophrenia. The International Journal of Psychiatry in Medicine [Internet]. 2008 Oct 2;38(1):103–12. Available from: https://doi.org/10.2190/PM.38.1.j

31.        Ellingrod VL, Miller DD, Taylor SF, Moline J, Holman T, Kerr J. Metabolic syndrome and insulin resistance in schizophrenia patients receiving antipsychotics genotyped for the methylenetetrahydrofolate reductase (MTHFR) 677C/T and 1298A/C variants. Schizophrenia Research [Internet]. 2008;98(1–3):47–54. Available from: https://europepmc.org/articles/pmc2271139?pdf=render

32.        Lee E, Leung C-M. Atypical antipsychotics and metabolic outcomes in Chinese patients: a comparison of olanzapine and risperidone. Journal of Clinical Psychopharmacology [Internet]. 2008;28(6):707–9. Available from: http://www.ncbi.nlm.nih.gov/pubmed/19011445

33.        Ojala K, Niskanen L, Tiihonen J, Paavola P, Putkonen A, Repo-Tiihonen E. Characterization of metabolic syndrome among forensic psychiatric inpatients. Journal of Forensic Psychiatry and Psychology [Internet]. 2008 Mar [cited 2020 Oct 5];19(1):33–51. Available from: https://www.tandfonline.com/doi/abs/10.1080/14789940701562519

34.        Yevtushenko OO, Cooper SJ, O’Neill R, Doherty JK, Woodside J v, Reynolds GP. Influence of 5-HT2C receptor and leptin gene polymorphisms, smoking and drug treatment on metabolic disturbances in patients with schizophrenia. The British journal of Psychiatry: the journal of mental science. 2008;192(6):424–8.

35.        Bai YM, Chen TT, Yang W-S, Chi Y-C, Lin C-C, Liou Y-J, et al. Association of adiponectin and metabolic syndrome among patients taking atypical antipsychotics for schizophrenia: A cohort study. Schizophrenia Research [Internet]. 2009 Jun 5 [cited 2020 Oct 4];111(1–3):1–8. Available from: https://linkinghub.elsevier.com/retrieve/pii/S0920996409001133

36.        Medved V, Kuzman MR, Jovanovic N, Grubisin J, Kuzman T. Metabolic syndrome in female patients with schizophrenia treated with second generation antipsychotics: a 3-month follow-up. Journal of Psychopharmacology (Oxford, England) [Internet]. 2009;23(8):915–22. Available from: http://www.ncbi.nlm.nih.gov/pubmed/18635691

37.        Patel JK, Buckley PF, Woolson S, Hamer RM, McEvoy JP, Perkins DO, et al. Metabolic profiles of second-generation antipsychotics in early psychosis: findings  from the CAFE study. Schizophrenia research. 2009;111(1–3):9–16.

38.        Fan X, Liu EY, Freudenreich O, Park JH, Liu D, Wang J, et al. Higher white blood cell counts are associated with an increased risk for metabolic syndrome and more severe psychopathology in non-diabetic patients with schizophrenia. Schizophrenia Research [Internet]. 2010 Oct 2;118(1–3):211–7. Available from: https://linkinghub.elsevier.com/retrieve/pii/S092099641001114X

39.        van Winkel R, Rutten BP, Peerbooms O, Peuskens J, van Os J, de Hert M. MTHFR and risk of metabolic syndrome in patients with schizophrenia. Schizophrenia Research [Internet]. 2010;121(1–3):193–8. Available from: http://www.ncbi.nlm.nih.gov/pubmed/20547447

40.        Kuzman MR, Medved V, Bozina N, Grubišin J, Jovanovic N, Sertic J. Association study of MDR1 and 5-HT2C genetic polymorphisms and antipsychotic-induced  metabolic disturbances in female patients with schizophrenia. The pharmacogenomics journal [Internet]. 2011;11(1):35–44. Available from: http://dx.doi.org/10.1038/tpj.2010.7

41.        Lee NY, Kim SH, Jung DC, Kim EY, Yu HY, Sung KH, et al. The prevalence of metabolic syndrome in Korean patients with schizophrenia receiving a monotherapy with aripiprazole, olanzapine or risperidone. Progress in Neuro-Psychopharmacology & Biological Psychiatry [Internet]. 2011;35(5):1273–8. Available from: http://www.ncbi.nlm.nih.gov/pubmed/21513765

42.        Ellingrod VL, Taylor SF, Dalack G, Grove TB, Bly MJ, Brook RD, et al. Risk factors associated with metabolic syndrome in bipolar and schizophrenia subjects treated with antipsychotics: the role of folate pharmacogenetics. Journal of Clinical Psychopharmacology [Internet]. 2012;32(2):261–5. Available from: https://europepmc.org/articles/pmc3622480?pdf=render

43.        Grover S, Aggarwal M, Dutt A, Chakrabarti S, Avasthi A, Kulhara P, et al. Prevalence of metabolic syndrome in patients with schizophrenia in India. Psychiatry Research [Internet]. 2012;200(2–3):1035–7. Available from: http://www.ncbi.nlm.nih.gov/pubmed/22503355

44.        Liou Y-J, Bai YM, Lin E, Chen J-Y, Chen T-T, Hong C-J, et al. Gene-gene interactions of the INSIG1 and INSIG2 in metabolic syndrome in  schizophrenic patients treated with atypical antipsychotics. The pharmacogenomics journal. 2012;12(1):54–61.

45.        Risselada AJ, Vehof J, Bruggeman R, Wilffert B, Cohen D, al Hadithy AF, et al. Association between HTR2C gene polymorphisms and the metabolic syndrome in patients using antipsychotics: A replication study [Internet]. Vol. 12, Pharmacogenomics Journal. Pharmacogenomics J; 2012 [cited 2020 Oct 4]. p. 62–7. Available from: https://pubmed.ncbi.nlm.nih.gov/20680028/

46.        Liou Y-J, Tsai S-J, Wang Y-C, Bai YM, Hong C-J. Genetic variants of microsomal triglyceride transfer protein (MTTP) are associated  with metabolic syndrome in schizophrenic patients treated with atypical  antipsychotics. Journal of clinical psychopharmacology. 2013;33(3):313–8.

47.        Lott SA, Burghardt PR, Burghardt KJ, Bly MJ, Grove TB, Ellingrod VL. The influence of metabolic syndrome, physical activity and genotype on catechol-O-methyl transferase promoter-region methylation in schizophrenia. The Pharmacogenomics Journal [Internet]. 2013;13(3):264–71. Available from: https://www.nature.com/articles/tpj20126.pdf

48.        Miller BJ, Mellor A, Buckley P. Total and Differential White Blood Cell Counts, High-Sensitivity C-Reactive Protein, and the Metabolic Syndrome in Non-Affective Psychoses. Brain, behavior, and immunity [Internet]. 2013 Oct 2;31:82–9. Available from: https://www.ncbi.nlm.nih.gov/pmc/articles/PMC5579743/

49.        Roffeei SN, Mohamed Z, Reynolds GP, Said MA, Hatim A, Mohamed EHM, et al. Association of FTO, LEPR and MTHFR gene polymorphisms with metabolic syndrome in schizophrenia patients receiving antipsychotics. Pharmacogenomics [Internet]. 2014;15(4):477–85. Available from: https://pdfs.semanticscholar.org/4a03/63b2eb9ce1d8d80f8362aa93116d89ac172d.pdf

50.        Lin EC-L, Shao W-C, Yang H-J, Yen M, Lee S-Y, Wu P-C, et al. Is abnormal non-high-density lipoprotein cholesterol a gender-specific predictor for metabolic syndrome in patients with schizophrenia taking second-generation antipsychotics? Metabolic Brain Disease [Internet]. 2015;30(1):107–13. Available from: http://www.ncbi.nlm.nih.gov/pubmed/25034455

51.        Saatcioglu O, Kalkan M, Fistikci N, Erek S, Kilic KC. Relationship Between Metabolic Syndrome and Clinical Features, and Its Personal-Social Performance in Patients with Schizophrenia. Psychiatric Quarterly [Internet]. 2016 Jun 1 [cited 2020 Oct 4];87(2):265–80. Available from: https://link.springer.com/article/10.1007/s11126-015-9384-0

52.        Yang CY, Lo SC, Peng YC. Prevalence and Predictors of Metabolic Syndrome in People With Schizophrenia in Inpatient Rehabilitation Wards. Biological Research for Nursing [Internet]. 2016 Oct 1 [cited 2020 Oct 4];18(5):558–66. Available from: http://journals.sagepub.com/doi/10.1177/1099800416653184

53.        Kraal AZ, Ward KM, Ellingrod VL. Sex Differences in Antipsychotic Related Metabolic Functioning in Schizophrenia  Spectrum Disorders. Psychopharmacology bulletin. 2017;47(2):8–21.

54.        Chen VC-H, Chen C-H, Chiu Y-H, Lin T-Y, Li F-C, Lu M-L. Leptin/Adiponectin ratio as a potential biomarker for metabolic syndrome in patients  with schizophrenia. Psychoneuroendocrinology. 2018;92:34–40.

55.        Larsen JR, Svensson CK, Vedtofte L, Jakobsen ML, Jespersen HS, Jakobsen MI, et al. High prevalence of prediabetes and metabolic abnormalities in overweight or obese schizophrenia patients treated with clozapine or olanzapine. CNS Spectrums [Internet]. 2019 Aug 1 [cited 2021 Jan 6];24(4):441–52. Available from: https://pubmed.ncbi.nlm.nih.gov/30596361/

56.        Dehelean L, Romosan AM, Manea MM, Papava I, Andor M, Romosan RS. The Metabolic Syndrome in Outpatients with Psychosis: a Comparative Study Between Long Acting Injectable Olanzapine and Risperidone. Acta Endocrinologica (Bucharest) [Internet]. 2019 Oct 2;15(3):342–8. Available from: https://www.ncbi.nlm.nih.gov/pmc/articles/PMC6992390/

57.        Iruretagoyena B, Castañeda CP, Undurraga J, Nachar R, Mena C, Gallardo C, et al. High prevalence of metabolic alterations in Latin American patients at initial  stages of psychosis. Early intervention in psychiatry. 2019;13(6):1382–8.

58.        Ventriglio A, Baldessarini RJ, Vitrani G, Bonfitto I, Cecere AC, Rinaldi A, et al. Metabolic Syndrome in Psychotic Disorder Patients Treated With Oral and Long-Acting Injected Antipsychotics. Frontiers in Psychiatry [Internet]. 2019 Jan 16 [cited 2020 Oct 4];9(JAN):744. Available from: https://www.frontiersin.org/article/10.3389/fpsyt.2018.00744/full

59.        Bai YM, Chen T-T, Liou Y-J, Hong C-J, Tsai S-J. Association between HTR2C polymorphisms and metabolic syndrome in patients with schizophrenia treated with atypical antipsychotics. Schizophrenia Research [Internet]. 2011 Oct 2;125(2):179–86. Available from: http://www.sciencedirect.com/science/article/pii/S0920996410016749

**
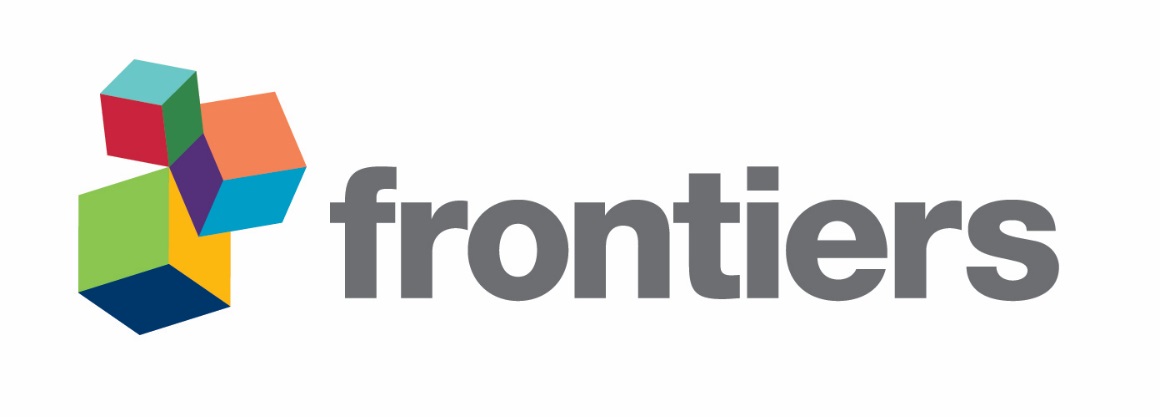
**
